# Effectiveness and Moderators of an Internet-Based Mobile-Supported Stress Management Intervention as a Universal Prevention Approach: Randomized Controlled Trial

**DOI:** 10.2196/22107

**Published:** 2021-12-22

**Authors:** David Daniel Ebert, Marvin Franke, Anna-Carlotta Zarski, Matthias Berking, Heleen Riper, Pim Cuijpers, Burkhardt Funk, Dirk Lehr

**Affiliations:** 1 Department of Sport and Health Sciences Technical University of Munich Munich Germany; 2 Clinical Psychology and Psychotherapy, Friedrich-Alexander University Erlangen-Nuremberg Erlangen Germany; 3 Department of Clinical, Neuro and Developmental Psychology, Amsterdam Public Health Research Institute, Vrije Universiteit Amsterdam Amsterdam Netherlands; 4 Department of Health Psychology and Applied Biological Psychology, Leuphana University of Lueneburg Lueneburg Germany

**Keywords:** stress management intervention, universal prevention, occupational health, moderators

## Abstract

**Background:**

Emerging evidence indicates the effectiveness of internet-based mobile-supported stress management interventions (iSMIs) in highly stressed employees. It is yet unclear, however, whether iSMIs are also effective without a preselection process in a universal prevention approach, which more closely resembles routine occupational health care. Moreover, evidence for whom iSMIs might be suitable and for whom not is scarce.

**Objective:**

The aim of this study was to evaluate the iSMI GET.ON Stress in a universal prevention approach without baseline inclusion criteria and to examine the moderators of the intervention effects.

**Methods:**

A total of 396 employees were randomly assigned to the intervention group or the 6-month waiting list control group. The iSMI consisted of 7 sessions and 1 booster session and offered no therapeutic guidance. Self-report data were assessed at baseline, 7 weeks, and at 6 months following randomization. The primary outcome was perceived stress. Several a priori defined moderators were explored as potential effect modifiers.

**Results:**

Participants in the intervention group reported significantly lower perceived stress at posttreatment (*d*=0.71, 95% CI 0.51-0.91) and at 6-month follow-up (*d*=0.61, 95% CI 0.41-0.81) compared to those in the waiting list control group. Significant differences with medium-to-large effect sizes were found for all mental health and most work-related outcomes. Resilience (at 7 weeks, *P*=.04; at 6 months, *P*=.01), agreeableness (at 7 weeks, *P*=.01), psychological strain (at 6 months, *P*=.04), and self-regulation (at 6 months, *P*=.04) moderated the intervention effects.

**Conclusions:**

This study indicates that iSMIs can be effective in a broad range of employees with no need for preselection to achieve substantial effects. The subgroups that might not profit had extreme values on the respective measures and represented only a very small proportion of the investigated sample, thereby indicating the broad applicability of GET.ON Stress.

**Trial Registration:**

German Clinical Trials Register DRKS00005699; https://www.drks.de/DRKS00005699

## Introduction

Occupational stress is a major public health problem, with high prevalence in western countries [1-3]. It is associated with a range of adverse consequences, including an increased risk for coronary disease [4,5], mental health problems [6,7], and sleeping problems [8]. Moreover, stress-related productivity loss, absenteeism, presentism, and health care usage lead to substantial economic costs [9]. Although manifold meta-analytic evidence exists for the effectiveness of psychological face-to-face stress management interventions for employees [10,11], the high prevalence of stress demands for highly cost-effective and scalable solutions. Internet-based mobile-supported stress management interventions (iSMIs) can be a great solution as they can offer several benefits such as (1) their accessibility at any time and place, (2) the possibility for participants to work and review materials at their own pace, and (3) their high potential for scalability [12]. In theory, only a small increase in resources is required for reaching a greater proportion of the eligible population. However, the real costs of iSMIs are greatly linked to the amount of guidance delivered by professional support [13], thereby limiting the possible reach of these interventions. Therefore, unguided interventions are an important puzzle piece to combat the high prevalence of stress.

The most recent meta-analysis on iSMIs found overall significant effectiveness (*d*=0.43, 95% CI 0.31-0.54) with small-to-medium effect sizes, but substantial heterogeneity between studies [14]. Subgroup analyses found that guided interventions (*d*=0.64, 95% CI 0.50-0.79; n=7) were significantly more effective than unguided interventions (*d*=0.33, 95% CI 0.20-0.46; n=18). Yet, the most effective iSMI GET.ON Stress yielded high effect sizes even when delivered in an unguided format [13], with an effect size (*d*=0.96, 95% CI 0.70-1.21) significantly exceeding that reported in the overall sample. In regard to the often cited replication crisis in Psychology [15] and other fields [16-18], it is crucial to evaluate if these effect sizes can withhold further inspection. Moreover, replicating these studies can help to address their shortcomings and add to the existing literature.

The first shortcoming relates to the evaluation in selected samples. Evidence for the best evaluated iSMI GET.ON Stress [13,19-25] is based on trials in which participants have been preselected based on the high baseline score in perceived stress (Perceived Stress Scale [PSS-10] ≥22) [26,27]. Using a broad range of eligibility criteria, however, can limit the real-life applicability of the results [28], as in routine preventive occupational health care, iSMIs are usually offered to a wide range of participants. Therefore, it is crucial to investigate treatment effects without preselection in a universal prevention approach [29]. This approach has not only theoretical but also practical advantages: while reaching a wider proportion of the working population, it reaches those that benefit from selected and indicated prevention without the cost for screening them specifically [30,31]. Additionally, it allows the inclusion of adults with lower symptom severity who are still motivated to improve their conditions, which might be a better indicator for their readiness for health behavior change [32].

The second shortcoming relates to the lack of moderator analyses. Even though internet-based intervention research is rapidly growing, empirical data on the moderators of internet-based mobile-supported interventions [33-39] and, in particular, internet occupational health interventions are scarce [34]. Furthermore, randomized controlled trials (RCTs) are usually powered to detect effects on the primary outcome and hence, underpowered to reliably test moderator hypotheses [40]. With reported numbers needed to treat of 5.43 for unguided iSMIs (and 4.20 in the overall sample) [14], it is clear, however, that a substantial proportion of the participants do not profit yet from taking part in an intervention. Therefore, identifying the moderators of iSMI effects is crucial for at least 3 reasons: (1) knowing who likely profits from the intervention helps to identify relevant populations, (2) knowing which patients are unlikely to profit from the treatment helps to prevent wasting resources and provides valuable information on where custom-tailoring such interventions to subgroups of patients is necessary, and (3) a better knowledge regarding who is (un)likely to profit from these interventions helps to identify mechanisms of change that are relevant for these interventions [41]. Therefore, the aim of this study was to evaluate the effects of the iSMI GET.ON Stress as a universal prevention approach, without preselecting participants based on symptom severity. Moreover, in an exploratory approach, a broad range of a priori selected potential effect modifiers were tested in a sample adequately powered for moderator analyses.

## Methods

### Design

**Figure 1:**
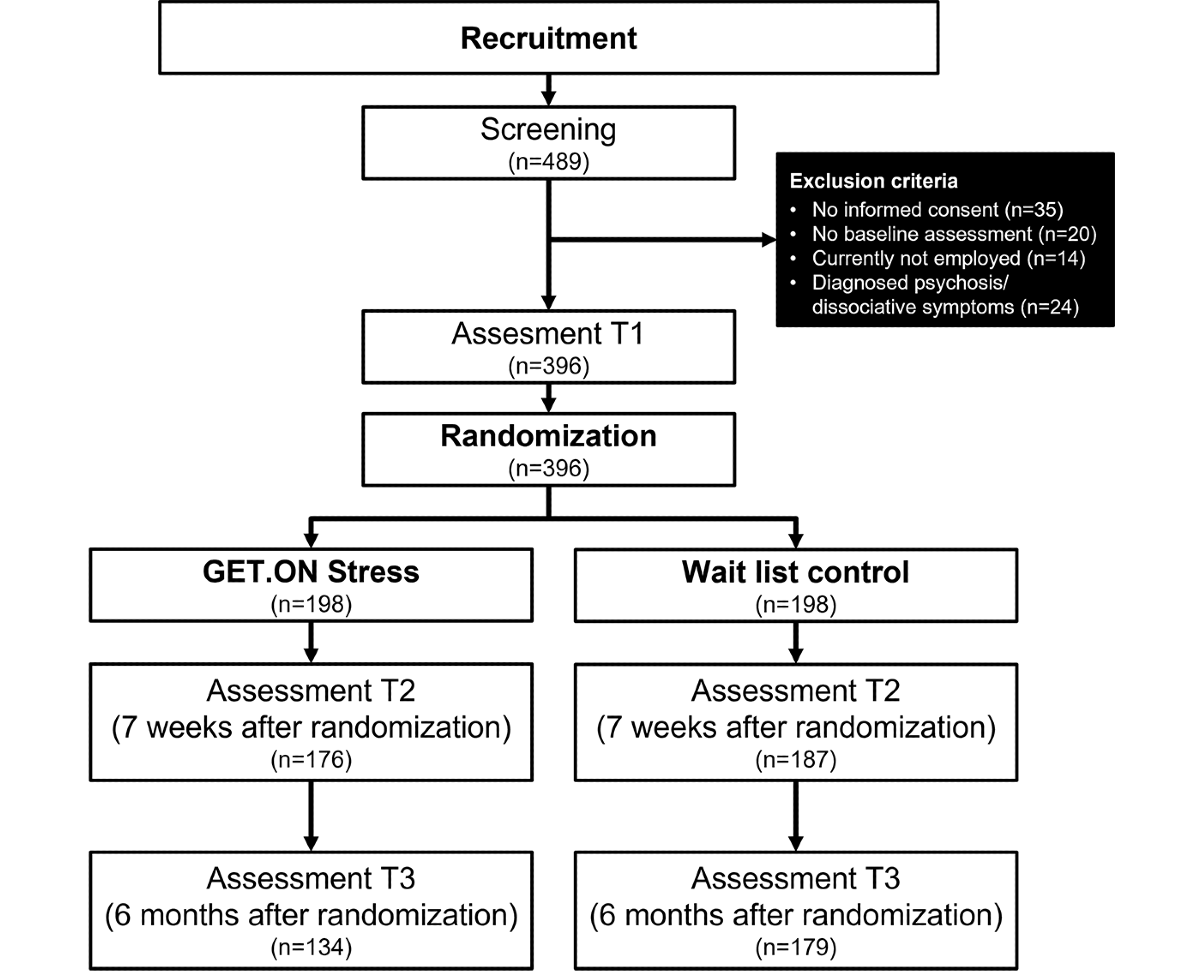
Flow of the study. T1: baseline; T2: at 7 weeks posttreatment; T3: at 6 months posttreatment.

A two-armed RCT (N=396) was conducted comparing a self-guided iSMI (GET.ON Stress) condition (intervention group [IG]) and a waiting list control (WLC) condition, with both conditions having full access to treatment as usual. Participants were recruited via the occupational health program of a large health insurance company in Germany (BARMER) in a way that mimics the intended implementation of the intervention in routine practice in the future, that is, by offering the intervention in a public occupational mental health approach to the general working population in a web-based setting. Recruitment was directed at the general working population and not restricted to members of the health care insurance company. Recruitment occurred mainly through reports in the membership magazine of the insurance company, and the insurance company’s occupational health management workers informed human resource departments of collaborating small- and medium-sized companies about the possibility for their employees to participate in the trial. Assessments took place at baseline (T1), at posttreatment (7 weeks, T2), and at 6 months (T3; see Figure 1 for a detailed overview of assessments). This study was approved by the University of Marburg ethics committee.

Interested individuals received an email with detailed information about the study procedures and an invitation to take part in the study. If they were interested, they were asked to complete a web-based screening questionnaire. Individuals meeting the eligibility criteria completed the baseline assessment and were invited to complete the informed consent form. Once the full written informed consent was received, participants entered the study and were randomly allocated to 1 of the 2 study conditions. Randomization took place at a ratio of 1:1. An independent third party who did not have any information about the participants performed the allocation. Randomization was carried out using an automated computer-based random integer generator (randlist). Other researchers could not bias the randomization process since participants were randomized in the order of the incoming informed consent form. Participants were not blinded to study conditions. During the randomization process, the allocation was concealed from the participants, researchers involved in recruitment, and the study administration team.

### Eligibility Criteria

Inclusion and exclusion criteria were kept to a minimum to mimic routine conditions as closely as possible. Participants were included who (1) were 18 years and older, (2) were currently employed, (3) had internet access, and (4) provided a valid email address. Individuals were only excluded if they were at risk of suicide (indicated by a score of 2 or higher on the Beck Depression Inventory Suicide Item [42]) or if they self-reported to have been diagnosed with psychosis or dissociative symptoms. Besides that, there were no restraints for participants, for example, concerning low or high stress, depression, or other symptom severity criteria. 

### Intervention

As this study evaluates an intervention that was evaluated in an indicated prevention context before, intervention content and delivery methods mirror those in related studies [20]. The training GET.ON Stress is based on Lazarus’ transactional model of stress [43]. According to this model, there are 2 coping strategies to deal with stress. One is a problem-oriented approach in changing the situation. The other approach focusses on situations where only the evaluation of the situation can be adjusted; this is called emotional regulation. Therefore, the training focuses on strategies for systematic problem-solving as well as emotion regulation. The intervention consists of 7 regular sessions and 1 booster session (provided 4 weeks after the last regular session was completed). The program starts with psychoeducation about stress (session 1), which guides participants to understand and find coping strategies for common problem situations. Based on their personal stressors, participants are asked to record their goals and motivation for the training. This is followed by 2 sessions focusing on problem-solving skills (sessions 2-3) where participants are guided through a 6-step method based on problem-solving therapy [44]. The personal plan will be implemented between the sessions before participants are asked to redo the exercise with the same or a different problem. The next part of the training (sessions 4-6) is based on the Affect Regulation training [45,46]. Each session focusses on 1 emotion regulation strategy: muscle and breathing relaxation, acceptance of emotions, as well as effective self-support with 15-minute audio files guiding users through the different exercises. Participants are encouraged to redo the exercises between sessions on a daily basis. The regular training is completed by a session guiding the creation of a “Plan for the future” (session 7), in which users are asked to review their progress and develop strategies according to their personal stress indicators in the future. Moreover, they are guided to write a letter to their future self. The booster session reviews the essential content of the intervention and allows participants to reassess their goals and plans for the future. Additionally, participants could choose from an array of elective modules in sessions 2 to 6 addressing common stress-related topics such as time management, sleep hygiene, or social support. Participants were advised to complete 1-2 sessions per week.

Across sessions, the training was composed of interactive education, audio and video files, and additional downloadable material. Additionally, testimonials offered impulses and examples concerning the exercises. The intervention is based on responsive design, meaning that it could be completed on a desktop computer, tablet, or smartphone. Through providing multiple choices among various response options, the content of the intervention was automatically tailored to the specific needs and interests of the individual participants. To integrate the newly acquired knowledge into daily life, homework assignments and behavioral planning were a crucial part of the training. Web-based diaries were also offered. The training was not accompanied by therapeutic guidance. However, the participants could opt for automatic text messages on their mobile phones choosing either a light (1 text message every other day) or intensive support (2-3 text messages per day). Text messages focused on ultrabrief exercises to be carried out in daily life routine, aiming to facilitate transfer from training into real life. 

### Outcomes

All measures were assessed through web-based self-report questionnaires and are described below. The assessment took place on a secured web-based system with 256-bit AES encryption. All outcomes were published a priori in detail in the trial register. The primary outcome was the perceived level of stress as measured by the German version of the PSS-10 [26,27] since it is based on Lazarus’ transactional model of stress. The 10 items were answered on a 5-point Likert scale (ranging from 0=never to 4=very often) referring to the past week. Owing to its sum score, the total scale scores range from 0 to 40. Cronbach alpha was .83 at T1, .91 at T2, and .91 at T3 in this study.

Secondary outcomes included measurement of mental health and work-related outcomes. Among those concerning mental health, the following outcomes were measured using specified scales: depression, using the German adaptation of the Center for Epidemiological Studies’ Depression Scale (15 items, range 0-45; *α*=.94) [47,48]; mental health–related quality of life, using the Assessment of Quality of Life (8 items, range 8-41; *α*=.84) [49]; resilience, using the Connor-Davidson Resilience Scale (10 items, range 10-50; *α*=.93) [50]; emotional exhaustion, using the subscale of the Maslach-Burnout-Inventory (5 items, range 1-6; *α*=.93) [51]; mental well-being, using the Warwick-Edinburgh Mental Wellbeing Scale (14 items, range 14-84; *α*=.96) [52]; and psychological well-being, using the 5-item World Health Organization Well-Being-Index (5 items, range 0-100; *α*=.91) [53]. The following work-related outcomes were assessed: effort-reward imbalance, using the Effort-Reward-Imbalance Questionnaire-Short Form (10 items, *α*=.84) [54]; occupational self-efficacy, using the Occupational Self-Efficacy Scale (6 items, range 6-36; *α*=.93) [55]; work engagement, using the Utrecht Work Engagement Scale (9 items, range 0-6; *α*=.97) [56]; productivity loss, using the Work-Limitations-Questionnaire (8 items, *α*=.87) [57]; and absenteeism and presentism days, using the respective questions from the German version of the Trimbos and Institute of Medical Technology Assessment Cost Questionnaire for Psychiatry (2 items) [58].

In addition, the following variables were assessed to be tested as potential moderators: personality, using the Big-Five-Inventory (10 items, subscale range *α*=.20-.77) [59]; motivation for treatment, using the Psychotherapy Motivation Questionnaire-Short Form (4 items, *α*=.36) [60]; self-regulation competencies, using the Self-Regulation scale (10 items, range 10-40; *α*=.84) [61]; and general self-efficacy, using the General Self-Efficacy Scale (10 items, range 10-40; *α*=.90) [62].

### Power Analysis

The sample size of the study was optimized to detect potential moderator effects. Previous studies on the same intervention found effects on the primary outcome of perceived stress at T2 ranging from *d*=0.84 to *d*=0.93 [13,23,63] in highly stressed employees. Assuming potentially lower effects, owing to the inclusion of less impaired employees, of approximately *d*=0.60, we would have needed to include 90 participants. Given that simulation studies showed that the sample size required to detect an interaction effect of the same magnitude needs to be at least threefold to fourfold, compared to the sample sizes needed for the primary analyses [64], we included 396 participants. This sample allowed us not only to detect a small regression effect of f² with a power (1-ß) of 80% and an error probability (*α*) of .05 but also a small intergroup effect size of *d*=0.25 in a one-tailed test (calculated with G*Power).

### Statistical Methods

All analyses are reported following the Consolidated Standards of Reporting Trials guidelines for reporting parallel group randomized trials [65]. Therefore, following the intention-to-treat principle, missing data were handled with multiple imputations [66], using 100 estimations per missing value. Outcome levels of IG and WLC were compared at T2 and T3 by using analysis of covariance (ANCOVA), with baseline levels as covariates. Additionally, Cohen *d* with 95% CIs were calculated based on the imputed data set by comparing means and SDs of the 2 groups at the respective time points. Furthermore, the difference in means along with its 95% CIs was calculated. To improve interpretability, reliable change was calculated according to the method of Jacobson and Truax [67] as an indicator of the number of participants with treatment response. Participants were accredited a treatment response if their PSS-10 score differed more than SD 5.16 in T1-T2 and T1-T3. Besides, the number needed to treat to achieve 1 additional treatment response compared to the control group was calculated. To evaluate the effect of the intervention on the risk for symptom worsening, we also calculated the number of participants with a reliable deterioration of symptoms by using the Reliable Change Index and calculated the absolute and relative reduction of risk, including the respective 95% CIs.

### Moderator Analysis

Regression analyses were used to test baseline moderator X intervention condition interaction effects by using the SPSS macro PROCESS [68]. In case of a significant interaction effect, we applied the Johnson-Neyman technique [68,69], which tests the conditional effect of X on Y at different values along the continuum of the moderator and calculates transition points, where the effect changes between statistically significant and not significant (at the *α* level of significance). This allows to identify a “region of significance” of the effect, giving away at which values of the moderator a significant effect can be found. This warrants essential advantages against the pick-a-point approach in which mostly only 3 arbitrarily chosen values of the moderator are tested since even the widespread use of the mean as well as SD is highly sample-specific and can thus lead to wrong conclusions [68]. Moderation variables were neither standardized nor mean-centered since it makes no difference for the moderation effect [68] and can even harm the interpretation if dichotomous variables are altered before the analysis.

## Results

### Participants

The study flow is illustrated in Figure 1. All 396 participants provided data at T1. In the IG, 176 (88.8%) participants at T2 and 134 (67.7%) participants at T3 and in the WLC group, 187 (94.4%) participants at T2 and 179 (90.4%) participants at T3 completed the assessment. The demographic characteristics of participants can be found in Table 1. The average age of the participants was 41.76 (SD 10.09) years. The sample was predominantly female (302/396, 76.3%), married, or in a relationship (209/396, 52.8%), as well as highly educated (285/396, 72%). Most participants were employed full-time (296/396, 74.7%), in a permanent employment relationship (306/396, 77.3%), and nearly half of them held a management function (169/396, 42.7%). Only a small portion (35/396, 8.8%) was self-employed. The average working experience was 17.58 (SD 10.36) years and the participants’ jobs were in various working sectors, with the majority in the economy (97/396, 24.5%) or social (79/396, 19.9%) sector. Only a small percentage had prior experience with health training (55/396, 13.9%). Having received psychotherapy in the past was stated by 147 (37.1%) participants, whereas only 35 (8.8%) indicated that they were currently receiving psychotherapy. Table 2 and Table 3 summarize the mean (SD) for the IG and WLC. Concerning the primary outcome at T1, participants had an average value of 22.65 (SD 5.63) on the PSS, thereby indicating a moderate stress level.

**Table 1:** Baseline characteristics of the study population (N=396).

Characteristics		All participants (N=396)	Intervention group (n=198)	Waiting list control group (n=198)
**Sociodemographic characteristics**				
	Age (years), mean (SD)	41.76 (10.09)	41.96 (10.34)	41.56 (9.87)
	Gender, female, n (%)	302 (76.3)	154 (77.8)	148 (74.7)
	Married or in a relationship, n (%)	209 (52.8)	106 (53.5)	103 (52)
	Having kids, n (%)	185 (46.7)	91 (46)	94 (47.5)
	West Germany, n (%)	351 (88.6)	176 (88.9)	175 (88.4)
**Ethnicity, n (%)**				
	Caucasian/White	319 (80.6)	161 (81.3)	158 (79.8)
	Asian	9 (2.3)	4 (2)	5 (2.5)
	Hispanic	2 (0.5)	2 (1)	0 (0)
	Prefer not to say	66 (16.7)	31 (15.7)	35 (17.7)
**Education, n (%)**				
	Low	17 (4.3)	8 (4)	9 (4.5)
	Middle	94 (23.7)	52 (26.3)	42 (21.2)
	High	285 (72)	138 (69.7)	147 (74.2)
**Working characteristics**				
	Full-time, n (%)	296 (74.7)	144 (72.7)	152 (76.8)
	Part-time, n (%)	93 (23.5)	50 (25.3)	43 (21.7)
	On sick leave, n (%)	7 (1.8)	4 (2)	3 (1.5)
	Management function, n (%)	169 (42.7)	87 (43.9)	82 (41.4)
	Work experience years, mean (SD)	17.58 (10.36)	17.62 (10.48)	17.54 (10.28)
**Employment status, n (%)**				
	Permanent	306 (77.3)	158 (79.8)	148 (74.7)
	Temporary	42 (10.6)	17 (8.6)	25 (12.6)
	Self-employed	35 (8.8)	15 (7.6)	20 (10.1)
	Other	13 (3.3)	8 (4)	5 (2.5)
**Working sectors, n (%)**				
	Economy	97 (24.5)	45 (22.7)	52 (26.3)
	Service	59 (14.9)	27 (13.6)	32 (16.2)
	Social	79 (19.9)	46 (23.2)	33 (16.7)
	Health	62 (15.7)	32 (16.2)	30 (15.2)
	Information technology	44 (11.1)	21 (10.6)	23 (11.6)
	Other	55 (13.9)	27 (13.6)	28 (14.1)
**Gross annual income (in €), n (%)**				
	Low income	89 (22.5)	37 (18.7)	52 (26.3)
	Middle income	134 (33.8)	72 (36.4)	62 (31.3)
	High income	123 (31.1)	61 (30.8)	62 (31.3)
	Not reported	50 (12.6)	28 (14.1)	22 (11.1)
**Experience, n (%)**				
	Previous health training	55 (13.9)	30 (15.2)	25 (12.6)
	Previous psychotherapy	147 (37.1)	73 (36.9)	74 (37.4)
	Current psychotherapy	35 (8.8)	16 (8.1)	19 (9.6)

**Table 2:** Means and standard deviations for the intention-to-treat sample at pretreatment.^a^

Outcome			Intervention group (n=198)					Waiting list control group (n=198)		
			Mean (SD)		Range		Mean (SD)			Range
**Primary outcome**										
	PSS^b^	22.39 (5.49)		8-39		22.91 (5.77)			6-37	
**Mental health**										
	CES-D^c^	16.02 (7.87)		1-43		16.44 (7.55)			1-38	
	AQoL8D-MH^d,e^	0.26 (0.11)		0.05-0.61		0.25 (0.12)			0.06-0.56	
	CD-RISC^e,f^	22.14 (6.63)		1-38		21.29 (7.00)			2-40	
	MBI-EE-D^g^	4.44 (0.85)		1.8-6		4.43 (0.91)			1.6-6.0	
	WEMWBS^e,h^	44.36 (7.51)		22-63		42.71 (7.97)			24-62	
	WHO-5^e,i^	35.09 (16.16)		0-80		35.05 (16.94)			0-96	
**Work-related outcomes**										
	ERI-S ratio^j^	1.34 (0.53)		0.42-3.62		1.30 (0.39)			0.29-3.09	
	OSES^e,k^	23.60 (6.53)		7-36		23.84 (6.35)			6-36	
	Absenteeism days	5.54 (10.75)		0-66		3.62 (7.97)			0-54	
	Presentism days	11.63 (13.50)		0-66		12.73 (15.93)			0-66	
	UWES^e,l^	3.35 (1.18)		0.33-5.89		3.18 (1.27)			0-5.67	
	WLQ^m^ productivity loss	8.44 (5.38)		0-22.82		8.36 (5.14)			0-24.53	

^a^Missing data imputed by multiple imputation.

^b^PSS: Perceived Stress Scale.

^c^CES-D: Center for Epidemiological Studies’ Depression Scale.

^d^AQoL8D-MH: Assessment Quality of Life 8-Dimensions (mental health component).

^e^Higher scores indicate better outcomes.

^f^CD-RISC: Connor-Davidson Resilience Scale.

^g^MBI-EE-D: Maslach Burnout Inventory (depletion subscale).

^h^WEMWBS: Warwick-Edinburgh Mental Wellbeing Scale.

^i^WHO-5: 5-item World Health Organization Well-Being Index.

^j^ERI-S: Effort-Reward-Imbalance Questionnaire-Short form.

^k^OSES: Occupational Self-Efficacy Scale.

^l^UWES: Utrecht Work Engagement Scale.

^m^WLQ: Work Limitations Questionnaire.

**Table 3:** Means and standard deviations for the intention-to-treat sample at posttreatment (T2) and 6-month follow-up (T3).

Outcome			Posttreatment at 7 weeks^a^					At 6-month follow-up^a^		
			Intervention group (n=198), mean (SD)		Waiting list control group (n=198), mean (SD)		Intervention group (n=198), mean (SD)			Waiting list control group (n=198), mean (SD)
**Primary outcome**										
	PSS^b^	16.27 (6.18)		21.22 (6.97)		15.73 (6.07)			19.94 (7.15)	
**Mental health**										
	CES-D^c^	11.26 (7.46)		15.52 (8.47)		11.45 (7.21)			15.37 (8.47)	
	AQoL8D-MH^d,e^	N/A^f^		N/A		0.36 (0.12)			0.29 (0.14)	
	CD-RISC^e,g^	24.41 (6.72)		20.79 (7.39)		N/A			N/A	
	MBI-EE-D^h^	3.94 (1.05)		4.31 (0.99)		3.73 (1.05)			4.27 (1.01)	
	WEMWBS^e,i^	49.28 (7.42)		44.19 (8.34)		50.55 (7.24)			44.02 (8.24)	
	WHO-5^e,j^	51.25 (18.02)		40.82 (19.92)		54.80 (15.64)			40.79 (20.12)	
**Work-related outcomes**										
	ERI-S ratio^k^	1.19 (0.51)		1.24 (0.45)		1.17 (0.40)			1.25 (0.42)	
	OSES^e,l^	26.21 (5.96)		23.89 (6.76)		N/A			N/A	
	Absenteeism days	N/A		N/A		4.03 (7.84)			3.22 (6.44)	
	Presentism days	N/A		N/A		8.88 (9.62)			11.75 (12.91)	
	UWES^e,m^	3.40 (1.22)		3.02 (1.33)		3.50 (1.03)			3.06 (1.25)	
	WLQ^n^ productivity loss	7.48 (5.67)		8.47 (5.50)		N/A			N/A	

^a^Missing data imputed by multiple imputation.

^b^PSS: Perceived Stress Scale.

^c^CES-D: Center for Epidemiological Studies’ Depression Scale.

^d^AQoL8D-MH: Assessment Quality of Life 8-Dimensions (mental health component).

^e^Higher scores indicate better outcomes.

^f^N/A: not applicable.

^g^CD-RISC: Connor-Davidson Resilience Scale.

^h^MBI-EE-D: Maslach Burnout Inventory (depletion subscale).

^i^WEMWBS: Warwick-Edinburgh Mental Wellbeing Scale.

^j^WHO-5: 5-item World Health Organization Well-Being Index.

^k^ERI-S: Effort-Reward-Imbalance Questionnaire-Short form.

^l^OSES: Occupational Self-Efficacy Scale.

^m^UWES: Utrecht Work Engagement Scale.

^n^WLQ: Work Limitations Questionnaire.

### Primary and Secondary Outcomes

Tables 4 and 5 display the results of the intention-to-treat analyses for the primary and secondary outcomes. The ANCOVA showed a significant group effect, indicating that participants in the IG had, compared to the WLC, significantly lower scores on the PSS-10 at T2 (*F*_1,393_=64.44, *P*<.001) and T3 (*F*_1,393_=46.91, *P*<.001) with medium-to-large between-group-effect sizes at T2 (Cohen *d*=0.71, 95% CI 0.51-0.91) and T3 (*d*=0.61, 95% CI 0.41-0.81). At posttreatment, significantly more participants in the IG (109/198, 55.1%) showed a reliable improvement on the PSS-10 compared to the WLC (44/198, 22.2%; *P*<.001). The number needed to treat to achieve 1 additional treatment response (reliable change) at posttreatment was 3.05 (95% CI 2.39-4.20). Significantly less participants experienced a symptom deterioration in the IG (10/198, 5.1%) compared to WLC (19/198, 9.6%), which reflects an absolute risk reduction of 4.55% (95% CI –0.567% to 9.658%) and a relative risk reduction of 47.88% (95% CI –10.307% to 74.888%).

**Table 4:** Results of the analysis of covariances and Cohen *d* for the primary and secondary outcome measures at posttreatment.

Outcome		At posttreatment after 7 weeks^a^ between-groups effect		
		Difference in means (95% CI)	Cohen *d* (95% CI)	ANCOVA^b^, *F(1,393*)
**Primary outcome**				
	PSS^c^	–4.66 (–5.80 to –3.51)	0.71 (0.51 to 0.91)	64.44^d^
**Mental health**				
	CES-D^e^	–4.00 (–5.27 to –2.74)	0.55 (0.35 to 0.75)	38.74^d^
	AQoL8D-MH^f,g^	N/A^h^	N/A	N/A
	CD-RISC^f,i^	2.97 (2.02 to 3.93)	0.54 (0.34 to 0.74)	37.27^d^
	MBI-EE-D^j^	–0.38 (–0.54 to –0.23)	0.47 (0.27 to 0.67)	23.60^d^
	WEMWBS^f,k^	4.05 (2.81 to 5.29)	0.50 (0.30 to 0.70)	41.31^d^
	WHO-5^f,l^	10.41 (7.16 to 13.66)	0.58 (0.38 to 0.78)	39.65^d^
**Work-related outcomes**				
	ERI-S^m^ ratio	–0.07 (–0.15 to 0.002)	0.21 (0.01 to 0.41)	3.65
	OSES^f,n^	2.49 (1.58 to 3.39)	0.51 (0.31 to 0.71)	29.30^d^
	Absenteeism days	N/A	N/A	N/A
	Presentism days	N/A	N/A	N/A
	UWES^f,o^	0.25 (0.08 to 0.43)	0.23 (0.03 to 0.42)	8.17^d^
	WLQ^p^	–1.01 (–2.07 to 0.05)	0.16 (–0.03 to 0.36)	3.51

^a^Missing data imputed by multiple imputation.

^b^Controlling for pretreatment scores (T1).

^c^PSS: Perceived Stress Scale.

^d^Significant at *P*<.001.

^e^CES-D: Center for Epidemiological Studies’ Depression Scale.

^f^Higher scores indicate better outcomes.

^g^AQoL8D-MH: Assessment Quality of Life 8-Dimensions (mental health component).

^h^N/A: not applicable.

^i^CD-RISC: Connor-Davidson Resilience Scale.

^j^MBI-EE-D: Maslach Burnout Inventory (depletion subscale).

^k^WEMWBS: Warwick-Edinburgh Mental Wellbeing Scale.

^l^WHO-5: 5-item World Health Organization Well-Being Index.

^m^ERI-S: Effort-Reward-Imbalance Questionnaire-Short form.

^n^OSES: Occupational Self-Efficacy Scale.

^o^UWES: Utrecht Work Engagement Scale.

^p^WLQ: Work Limitations Questionnaire.

**Table 5:** Results of the analysis of covariances and Cohen *d* for the primary and secondary outcome measures at posttest and 6-month follow-up.

Outcome			T3^a^ Between-groups effect				
			Difference in means (95% CI)		Cohen *d* (95% CI)		ANCOVA^b^*, F(1,393)*
**Primary outcome**							
	Perceived Stress Scale	–3.89 (–5.01 to –2.77)		0.61 (0.41 to 0.81)		46.9^c^	
**Mental health**							
	Center for Epidemiological Studies’ Depression Scale	–3.65 (–4.87 to –2.44)		0.52 (0.32 to 0.72)		34.88^c^	
	AQoL8D-MH^d,e^	0.06 (0.04 to 0.08)		0.52 (0.31 to 0.72)		32.09^c^	
	CD-RISC^d,f^	N/A		N/A		N/A	
	MBI-EE-D^g^	–0.56 (–0.73 to –0.39)		0.61 (0.41 to 0.81)		41.28^c^	
	WEMWBS^d,h^	5.63 (4.34 to 6.93)		0.66 (0.46 to 0.86)		73.25^c^	
	WHO-5^d,i^	13.99 (10.78 to 17.21)		0.76 (0.55 to 0.96)		73.32^c^	
**Work-related outcomes**							
	ERI-S^j^ ratio	–0.10 (–0.17 to –0.03)		0.77 (0.56 to 0.97)		7.53^k^	
	OSES^d,l^	N/A		N/A		N/A	
	Absenteeism days	0.48 (–0.91 to 1.87)		0.11 (–0.09 to 0.30)		0.46	
	Presentism days	–2.44 (–4.40 to –0.49)		0.13 (–0.07 to 0.33)		6.04^m^	
	UWES^d,n^	0.33 (0.15 to 0.50)		0.25 (0.05 to 0.45)		13.37^c^	
	WLQ^o^	N/A		N/A		N/A	

^a^Missing data imputed by multiple imputation.

^b^Controlling for pretreatment scores (T1).

^c^Significant at *P*<.001.

^d^Higher scores indicate better outcomes.

^e^AQoL8D-MH: Assessment Quality of Life 8-Dimensions (mental health component).

^f^CD-RISC: Connor-Davidson Resilience Scale.

^g^MBI-EE-D: Maslach Burnout Inventory (depletion subscale).

^h^WEMWBS: Warwick-Edinburgh Mental Wellbeing Scale.

^i^WHO-5: 5-item World Health Organization Well-being Index.

^j^ERI-S: Effort-Reward-Imbalance Questionnaire-Short form.

^k^Significant at *P*<.01.

^l^OSES: Occupational Self-Efficacy Scale.

^m^Significant at *P*<.05.

^n^UWES: Utrecht Work Engagement Scale.

^o^WLQ: Work Limitations Questionnaire.

Comparing reliable changes from baseline to 6 months, 53.5% (106/198) in the IG showed a reliable improvement in contrast to 36.4% (72/198) in the WLC (*P*<.001). The number needed to treat to achieve one additional reliable improvement at 6 months follow-up was 5.82 (95% CI 3.73-13.30). A reliable deterioration was only present in 2% (4/198) of the IG, which was significantly lesser (*P*<.05) than that in the WLC (13/198, 6.6%).

As shown in Tables 4 and 5, the ANCOVAs for mental health outcomes showed significant between-group effects for all outcomes at both assessment points on a *P*<.001 level in favor of the IG. At T2, all effect sizes were moderate ranging from *d*=0.47 for emotional exhaustion to *d*=0.58 for well-being according to the 5-item World Health Organization Well-Being Index. At T3, moderate-to-large effect sizes could be found ranging from *d*=0.52 for mental health to *d*=0.76 for well-being.

Concerning work-related outcomes, the results were less coherent. At T2, the ANCOVAs for work-related self-efficacy (*P*<.001) and work engagement (*P*=.004) showed significant between-group effects, with a small effect size for work engagement (*d*=0.23) and a moderate effect size for occupational self-efficacy (*d*=0.51). There was no significant difference between groups for effort-reward-ratio (*P*=.06) and productivity loss (*P*=.06). However, at T3, apart from absenteeism days (*P*=.50), all between-group effects were significant (effort-reward-imbalance T3, *P*=.006; presentism days T3, *P*=.01; work engagement T3, *P*<.001), with a large effect size of *d*=0.77 for effort-reward-imbalance and a small effect size of *d*=0.25 for work engagement and presentism days (*d*=0.13).

### Adherence

Of the 198 participants in the IG, 188 (95%) finished session 1. Session 2 was completed by 171 (86.4%), session 3 by 154 (77.8%), session 4 by 135 (68.2%), session 5 by 121 (61.1%), session 6 by 108 (54.5%), session 7 by 94 (47.5%), and the booster session by 66 (33.3%) individuals. On average, participants worked through 5.23 (SD 2.74) sessions representing 66% (5.23/8) of the intervention. A linear regression model controlling for baseline stress indicated that the number of completed sessions significantly predicted stress levels at T2 (*b*=–0.67, SE 0.14; *P*<.001; 95% CI –0.94 to –0.39) and T3 (*b*=–0.39, SE 0.14; *P*=.004; 95% CI –0.66 to –0.12). The regression coefficient suggested that the PSS value decreased 0.67 (T2) and respectively 0.39 (T3) points with each additional module completed.

### Moderator Analysis

**Figure 2:**
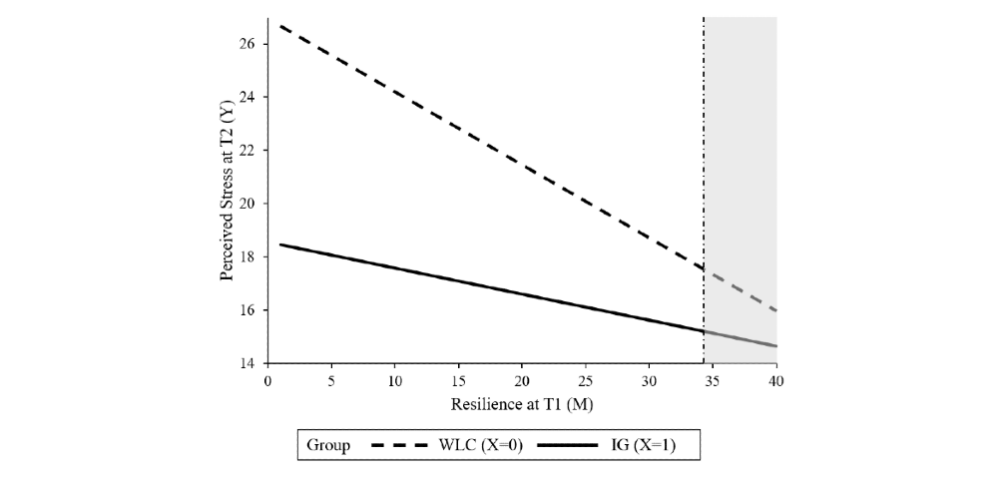
A visual representation of the moderation of the training effect (X) on perceived stress at T2 (Y) by resilience at T1 (M) accompanied with a visual representation of the area of significance according to the Johnson-Neyman technique. IG: intervention group; T1: baseline; T2: at 7 weeks posttreatment; WLC: waiting list control.

**Figure 3:**
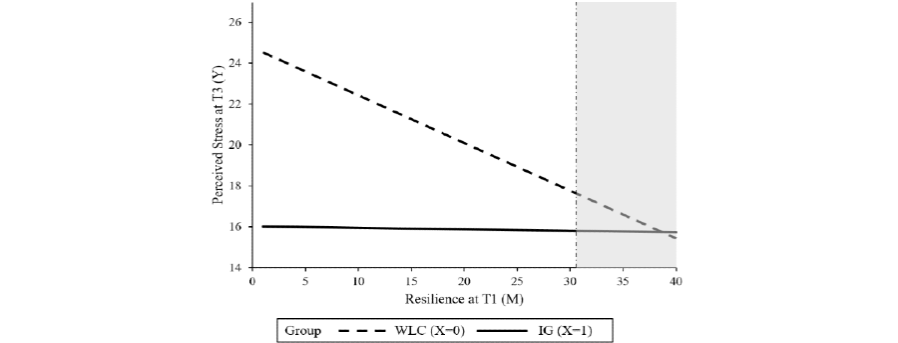
A visual representation of the moderation of the training effect (X) on perceived stress at T3 (Y) by resilience at T1 (M) accompanied with a visual representation of the area of significance according to the Johnson-Neyman technique. IG: intervention group; T1: baseline; T3: at 6 months posttreatment; WLC: waiting list control.

**Figure 4:**
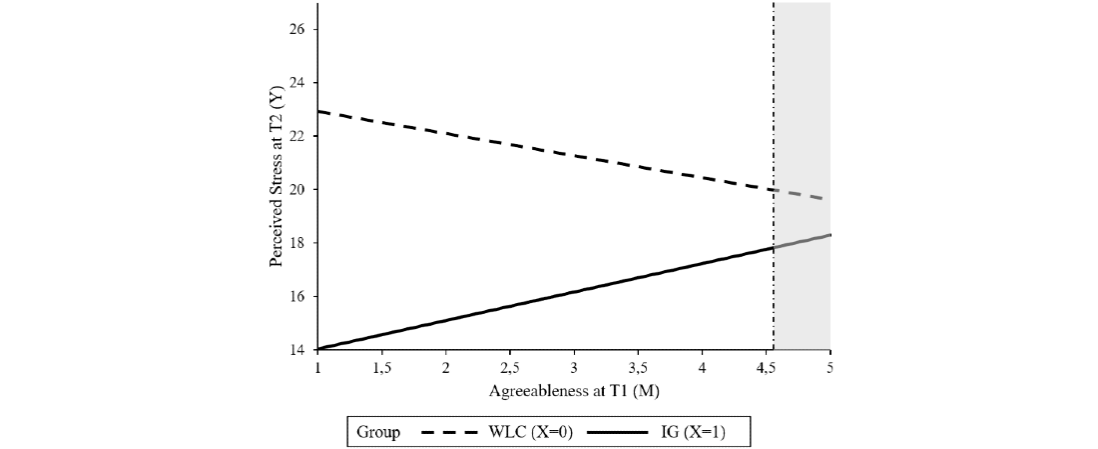
A visual representation of the moderation of the training effect (X) on perceived stress at T2 (Y) by agreeableness at T1 (M) accompanied with a visual representation of the area of significance according to the Johnson-Neyman technique. IG: intervention group; T1: baseline; T2: at 7 weeks posttreatment; WLC: waiting list control.

**Figure 5:**
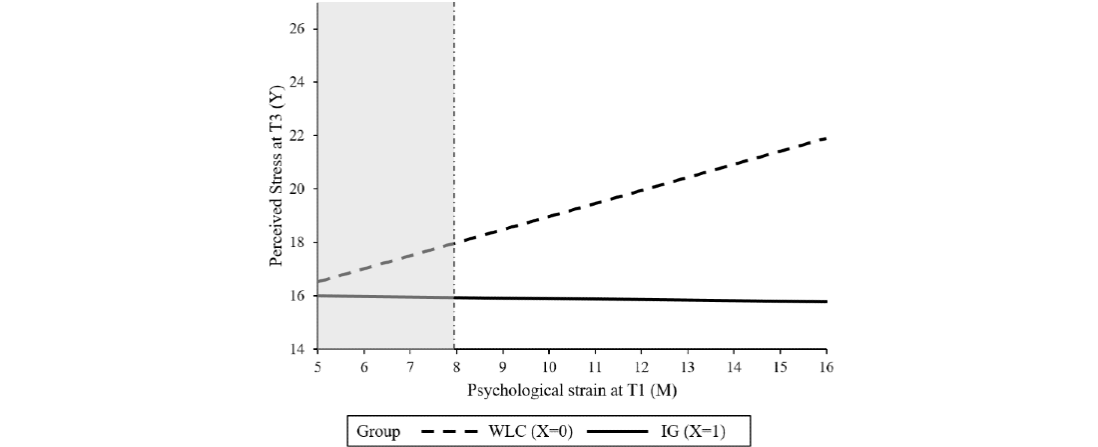
A visual representation of the moderation of the training effect (X) on perceived stress at T3 (Y) by psychological strain at T1 (M) accompanied with a visual representation of the area of significance according to the Johnson-Neyman technique. IG: intervention group; T1: baseline; T3: at 6 months posttreatment; WLC: waiting list control.

**Figure 6:**
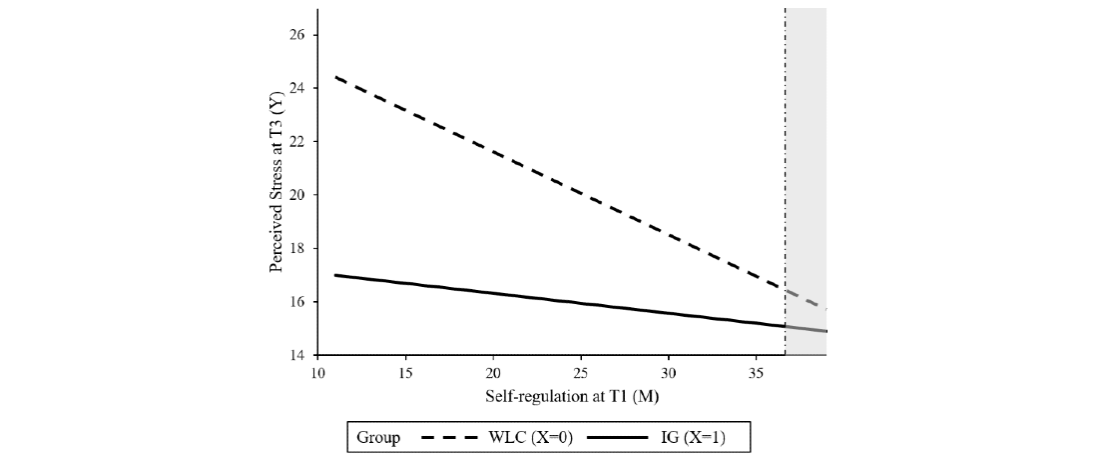
A visual representation of the moderation of the training effect (X) on perceived stress at T3 (Y) by self-regulation at T1 (M) accompanied with a visual representation of the region of significance according to the Johnson-Neyman technique. IG: intervention group; T1: baseline; T3: at 6 months posttreatment; WLC: waiting list control.

Baseline symptom severity was not found to be a significant moderator, indicating that the intervention can be effective, irrespective of the baseline level of perceived stress. Resilience, agreeableness, psychological strain, and self-regulation competencies significantly moderated the treatment outcome. Resilience moderated the intervention effect at T2 (*P*=.04) as well as at T3 (*P*=.01). The region of significance at T2 and T3 ranged from 1 to 34.28 and 1 to 30.63, respectively, indicating that there was no significant between-group effect for participants with higher levels of resilience at baseline. In total, 2.8% (11/396) of the sample had resilience scores >34.28; 8.8% (35/396) had scores >30.63. Agreeableness moderated the intervention effect on stress at T2 (*P*=.01) with a region of significance from 1 to 4.55, indicating no significant intervention effects within participants with a higher agreeableness score (2 out of 396 participants had agreeableness scores above 4.55). The intervention effect on stress at T3 was moderated by psychological strain (*P*=.04) and self-regulation (*P*=.04). No significant between-group effect was found for participants with a psychological strain score lower than 7.96 (19/396, 4.8%) or self-regulation scores higher than 33.57 (24/396, 6%). Tables S1-S4 in Multimedia Appendix 1 show the results of the moderation analyses whereas Figures 2-6 show a visual representation of the moderation accompanied by a visual representation of the region of significance.

No demographic variable (age, gender, relationship status, having kids), other work-related characteristics (full-time work, having a management function, work experience, employment status, working sector, gross annual income), as well as other symptom severity indicators were significantly associated with the intervention effects. *P* values of nonsignificant interaction effects ranged from *P*=.08 (T2) for gender to *P*=.99 for conscientiousness.

## Discussion

This study aimed to evaluate the effectiveness and moderators of treatment outcome of a self-guided iSMI as a universal prevention approach without an elaborative inclusion process based on baseline symptom severity. The results of our study indicate that the training was highly effective in reducing stress levels in the short term (*d*=0.71) and long term (*d*=0.61). Significant moderate-to-large effects were found for several secondary mental health and work-related outcomes, including outcomes such as occupational self-efficacy for which no evidence of iSMIs was yet available. Moreover, this study is one of the first to investigate moderator effects in iSMIs in an adequately powered sample. The intervention was suitable for a wide range of participants. Only a few moderators of the intervention effect were identified, indicating that employees with very high resilience, very low psychological strain, very high agreeableness, and very high self-regulation might not profit from the iSMI.

The effects in this study were lower than those in the 4 previously conducted RCTs that examined the same iSMI but in which participants were preselected (PSS>22 or PSS-4 ≥8). With therapeutic guidance, large effect sizes of *d*=0.83 at posttreatment (95% CI 0.58-1.08) were found compared to that of WLC [63], whereas with adherence-focused guidance [23], the between-group effects were *d*=0.79 (95% CI 0.54-1.04), and as a purely self-guided intervention in employees [13] or students [25], the effect size was *d*=0.96 (95% CI 0.70-1.21) or *d*=0.69 (95% CI 0.36-1.02), respectively. However, the CIs in this trial (95% CI 0.51-0.91) overlapped those of all previously conducted studies.

When comparing the results with those from unguided iSMIs not necessarily conducted in the work settings, the effect sizes found in this trial were larger than those found in a recent meta-analysis on the topic (*d*=0.33, 95% CI 0.20-0.46) [70] also when taking 95% CIs into account. Potential reasons for the higher effect sizes compared to other interventions might lie in the strong theoretical basis and the possibility to tailor the intervention to individual need or interest. An alternative explanation might be the strong focus of the intervention on supporting participants to implement health behavior changes in daily life routine. Although the study was powered to test moderation hypotheses adequately, and although we included a broad range of theoretically potential effect modifiers, only few baseline variables significantly moderated the intervention effects. Baseline perceived stress was not associated with intervention outcome, indicating that GET.ON Stress can also be effective in heterogeneous samples that are not preselected based on high baseline symptom severity. That said, it needs to be noted that the sample showed a substantial level of baseline impairment, indicated by a mean of 22.65 (SD 5.63) on the PSS. Despite being lower than that in previous studies that have been conducted on GET.ON Stress (PSS, 25.26-23.90), and although 40.3% (160/396) of the participants in this trial would have been excluded in previous trials, the average baseline symptom severity was still approximately 1 SD above the average perceived stress level in a large working population (mean 15.3 [SD 6.2]) [71]. This might indicate that the intervention might especially be attractive for employees who already experience a substantial stress level. Future studies should hence test whether the utilization of universal preventive approaches in employees can be further increased, for example, by utilizing acceptance-facilitating interventions [72-75] that are designed to reduce the barriers of intervention utilization, such as low perceived risk. Another possibility might be that the format is not adequate for employees with low perceived burden and interventions with even a lower threshold, for example, mobile apps, focusing on less burdensome behavior changes are necessary to reach this target group.

Resilience, psychological strain, agreeableness, and self-regulation moderated the treatment outcome. Our findings clearly show that the difference in the perceived stress between the IG and the WLC is dependent on the resilience score at baseline. This difference (in the perceived stress between the IG and the WLC) is greater in those that show a lower resilience at baseline, whereas the difference almost becomes nonexistent if the baseline resilience is very high. One potential explanation for this finding might be that very resilient participants manage to cope with perceived stress also in the control group without the help of an additional psychological intervention. Such an interpretation is in line with the theoretical framework around resilience assuming that resilience helps to deal with stressors [76,77]. Several systematic reviews show that high resilience might serve as a protective buffer against the development of psychopathological symptoms [78-82].

Interestingly, employees with low self-regulation profited to a much greater extent from participating compared to those with high self-regulation. This is in contrast to our a priori expectation as we assumed that self-regulation competencies, the ability of an individual to regulate and control their thoughts and behavior enabling them to adapt to a broad range of demands [83], might be a necessary prerequisite for making effective use of such self-help approaches, which require the self-regulated implementation of behavior changes. However, one explanation for the finding might be that participating in the iSMI helps employees with low-self regulation to effectively counteract the missing necessary competencies required to effectively implement health behavior changes in daily life that are needed in order to reduce perceived stress, whereas employees with high self-regulation might manage to realize these necessary self-regulatory tasks also without an additional intervention. Such an assumption of a compensatory effect is supported by the data in the control group showing very high perceived stress level at follow-up in the control group for those with low self-regulation in comparison to low perceived stress levels in the control group for those with high self-regulation. In the IG, the difference between low and high self-regulation is, however, not reflected in the major differences in perceived stress. Nonetheless, empirical studies are needed to confirm these assumptions.

The finding that the intervention was found to be ineffective in individuals with very low current psychological strain (<7.96) has relevant implications for the implementation of such approaches. If these results are confirmed in future studies, such interventions should not be used solely as a universal preventive intervention, for example, to increase protective factors that might help to cope with future stressors. Instead, such interventions should only be offered to employees experiencing at least a minimal level of psychological strain, as this might serve as an important source of motivation that is required for self-help approaches. However, our study was limited to a 6-months follow-up, and future studies are needed to investigate the potential protective effects in such populations regarding future stressors. This study also found agreeableness as a moderator, which has not emerged in the previous literature. Future research should investigate if this was a spurious finding or could lead to a new understanding of the interaction between personality traits and iSMIs.

This study also has some limitations to be considered. First, as usual with RCTs, there was a screening process preceding trial participation, that is, informed consent, which might have caused some applicants with lower motivation to drop out before the study began, potentially resulting in a more homogenous sample. Moreover, the exclusion of suicidal individuals limits the generalizability to such samples. Therefore, although this was a pragmatic trial and eligibility criteria were kept to a minimum to mimic routine care, intervention effects might be overestimated compared to the intervention under routine conditions. Second, one needs to keep in mind that the evidence generated by this study is based on an RCT, which brings a rather high structuring of participants and a high research attention with it, which is usually not the case in routine occupational care. Since the securing of commitment represents an adherence-promoting element in self-help interventions, it can be assumed that the effect sizes for pure self-guided interventions found in RCTs are significantly overestimated for what can be expected in occupational routine care [84] when no additional measures to increase adherence are applied. Hence, a clear concept for ensuring adherence such as through minimal guidance from a professional seems favorable, especially when considering that guided internet-based interventions have found to be superior over pure self-help interventions, both for stress-management [70] as well as other areas [85-87]. Preference should be in routine care, whenever possible, given to self-help approaches with at least some form of adherence-promoting guidance. Moreover, although the study was powered for moderator analyses, we did not stratify for extreme values on assessed moderator variables. Hence, some expressions on the investigated moderators, for example, very low stress levels, were probably not included in sufficient numbers so that a moderator analysis could have detected an existing effect. Further, the power was not sufficient to detect small moderator effects that may nevertheless be of clinical relevance. Since this is one of the first trials on iSMIs as well as SMIs, in general, investigating moderator effects, future empirical studies are needed to confirm our findings.

This study confirms GET.ON Stress to be effective in a heterogeneous sample of employees and to be applicable for a broad range of participant characteristics. Based on the available evidence, iSMIs should be implemented on a broad scale to reduce the adverse consequences of occupational stress.

## Appendix

Multimedia Appendix 1 Supplementary data.

Multimedia Appendix 2 CONSORT-eHEALTH checklist (V 1.6.1).

## Abbreviations

ANCOVA: analysis of covariance
IG: intervention group
iSMI: internet-based mobile-supported stress management intervention
PSS: perceived stress scale
RCT: randomized controlled trial
WLC: waiting list control
