# Supplementary material for: Effectiveness and Moderators of an Internet-Based Mobile-Supported Stress Management Intervention as a Universal Prevention Approach: Randomized Controlled Trial
Source: J Med Internet Res. 2021 Dec 22;23(12):e22107. doi: 10.2196/22107 (PMC8734929; doi:10.2196/22107)
Supplement: Multimedia Appendix 1 [file jmir_v23i12e22107_app1.docx]

**Table S1.** Results from a regression analysis examining the moderation of the training effect about the resilience score at T1 on perceived stress at T2 (R²=0.36) and T3 (R²=0.37) with the baseline stress level as a covariate

|  | T2 | | | |  | T3 | | | |
| --- | --- | --- | --- | --- | --- | --- | --- | --- | --- |
|  | Coefficient | SE | *t* (*df*) | *P* |  | Coefficient | SE | *t* (*df*) | *P* |
| Constant | 16.53 | 2.20 | 7.53 (391) | < .001 |  | 12.28 | 2.17 | 5.67 (391) | < .001 |
| Group (X) | –8.38 | 1.90 | –4.41 (391) | < .001 |  | –8.73 | 1.87 | –4.66 (391) | < .001 |
| CD-RISC at T1 (M) | –0.27 | 0.06 | –4.49 (391) | < .001 |  | -0.23 | 0.06 | –3.87 (391) | < .001 |
| Group x CD-RISC at T1 (XM) | 0.18 | 0.08 | 2.11 (391) | .04 |  | 0.23 | 0.08 | 2.74 (391) | .01 |
| PSS-10 at T1 (C) | 0.46 | 0.06 | 7.93 (391) | < .001 |  | 0.55 | 0.06 | 9.65 (391) | < .001 |

Abbreviations: CD-RISC=Connor-Davidson Resilience Scale; PSS-10=Perceived Stress Scale.

**Table S2.** Results from a regression analysis examining the moderation of the training effect about the agreeableness at T1 on perceived stress at T2 (R²=0.34) and T3 (R²=0.35) with the baseline stress level as a covariate

|  | T2 | | | |  | T3 | | | |
| --- | --- | --- | --- | --- | --- | --- | --- | --- | --- |
|  | Coefficient | SE | *t* (*df*) | *P* |  | Coefficient | SE | *t* (*df*) | *P* |
| Constant | 10.89 | 2.17 | 5.03 (391) | < .001 |  | 8.16 | 2.13 | 3.83 (391) | < .001 |
| Group (X) | –10.80 | 2.41 | –4.48 (391) | < .001 |  | –7.02 | 2.37 | –2.96 (391) | .003 |
| Agreeableness at T1 (M) | –0.83 | 0.49 | –1.68 (391) | .09 |  | -0.70 | 0.48 | –1.44 (391) | .15 |
| Group x Agreeableness at T1 (XM) | 1.89 | 0.72 | 2.63 (391) | .01 |  | 0.96 | 0.71 | 1.35 (391) | .18 |
| PSS-10 at T1 (C) | 0.57 | 0.05 | 10.90 (391) | < .001 |  | 0.61 | 0.05 | 11.93 (391) | < .001 |

Abbreviations: PSS-10=Perceived Stress Scale.

**Table S3.** Results from a regression analysis examining the moderation of the training effect about the psychological strain at T1 on perceived stress at T2 (R²=0.34) and T3 (R²=0.36) with the baseline stress level as a covariate

|  | T2 | | | |  | T3 | | | |
| --- | --- | --- | --- | --- | --- | --- | --- | --- | --- |
|  | Coefficient | SE | *t* (*df*) | *P* |  | Coefficient | SE | *t* (*df*) | *P* |
| Constant | 3.49 | 2.16 | 1.61 (391) | .11 |  | 0.91 | 2.12 | 0.43 (391) | .67 |
| Group (X) | 0.58 | 2.93 | 0.20 (391) | .84 |  | 2.00 | 2.86 | 0.70 (391) | .48 |
| Psychological strain at T1 (M) | 0.49 | 0.18 | 2.70 (391) | .007 |  | 0.49 | 0.18 | 2.78 (391) | .01 |
| Group x Psychological strain at T1 (XM) | –0.45 | 0.25 | –1.80 (391) | .072 |  | –0.51 | 0.24 | –2.08 (391) | .04 |
| PSS-10 at T1 (C) | 0.53 | 0.05 | 9.49 (391) | < .001 |  | 0.58 | 0.05 | 10.73 (391) | < .001 |

Abbreviations: PSS-10=Perceived Stress Scale.

**Table S4.** Results from a regression analysis examining the moderation of the training effect about the self-regulation at T1 on perceived stress at T2 (R²=0.34) and T3 (R²=0.36) with the baseline stress level as a covariate

|  | T2 | | | |  | T3 | | | |
| --- | --- | --- | --- | --- | --- | --- | --- | --- | --- |
|  | Coefficient | SE | *t* (*df*) | *P* |  | Coefficient | SE | *t* (*df*) | *P* |
| Constant | 16.01 | 3.08 | 5.19 (391) | < .001 |  | 15.21 | 3.00 | 5.06 (391) | < .001 |
| Group (X) | –9.01 | 3.21 | –2.81 (391) | .0052 |  | –9.99 | 3.13 | –3.20 (391) | .0015 |
| Self-regulation at T1 (M) | –0.25 | 0.09 | –2.69 (391) | .0074 |  | –0.31 | 0.09 | –3.39 (391) | .0008 |
| Group x Self-regulation at T1 (XM) | 0.17 | 0.12 | 1.38 (391) | .17 |  | 0.24 | 0.12 | 1.99 (391) | .04 |
| PSS-10 at T1 (C) | 0.51 | 0.06 | 9.18 (391) | < .001 |  | 0.56 | 0.05 | 10.24 (391) | < .001 |

Abbreviations: PSS-10=Perceived Stress Scale.
