# Supplementary material for: Effectiveness and Moderators of an Internet-Based Mobile-Supported Stress Management Intervention as a Universal Prevention Approach: Randomized Controlled Trial
Source: J Med Internet Res. 2021 Dec 22;23(12):e22107. doi: 10.2196/22107 (PMC8734929; doi:10.2196/22107)
Supplement: Multimedia Appendix 2 [file jmir_v23i12e22107_app2.pdf]

CONSORT-EHEALTH (V 1.6.1) - Submission/Publication Form

The CONSORT-EHEALTH checklist is intended for authors of randomized trials evaluating web-based and Internet-based applications/interventions, including mobile interventions, electronic games (incl multiplayer games), social media, certain telehealth applications, and other interactive and/or networked electronic applications. Some of the items (e.g. all subitems under item 5 - description of the intervention) may also be applicable for other study designs.

The goal of the CONSORT EHEALTH checklist and guideline is to be
a) a guide for reporting for authors of RCTs,
b) to form a basis for appraisal of an ehealth trial (in terms of validity)

CONSORT-EHEALTH items/subitems are MANDATORY reporting items for studies published in the Journal of Medical Internet Research and other journals / scientific societies endorsing the checklist.

Items numbered 1., 2., 3., 4a., 4b etc are original CONSORT or CONSORT-NPT (non-pharmacologic treatment) items.
Items with Roman numerals (i., ii, iii, iv etc.) are CONSORT-EHEALTH extensions/clarifications.

As the CONSORT-EHEALTH checklist is still considered in a formative stage, we would ask that you also RATE ON A SCALE OF 1-5 how important/useful you feel each item is FOR THE PURPOSE OF THE CHECKLIST and reporting guideline (optional).

Mandatory reporting items are marked with a red *.
In the textboxes, either copy & paste the relevant sections from your manuscript into this form - please include any quotes from your manuscript in QUOTATION MARKS,
or answer directly by providing additional information not in the manuscript, or elaborating on why the item was not relevant for this study.

YOUR ANSWERS WILL BE PUBLISHED AS A SUPPLEMENTARY FILE TO YOUR PUBLICATION IN JMIR AND ARE CONSIDERED PART OF YOUR PUBLICATION (IF ACCEPTED).
Please fill in these questions diligently. Information will not be copyedited, so please use proper spelling and grammar, use correct capitalization, and avoid abbreviations.

DO NOT FORGET TO SAVE AS PDF _AND_ CLICK THE SUBMIT BUTTON SO YOUR ANSWERS ARE IN OUR DATABASE !!!

Citation Suggestion (if you append the pdf as Appendix we suggest to cite this paper in the caption):
Eysenbach G, CONSORT-EHEALTH Group
CONSORT-EHEALTH: Improving and Standardizing Evaluation Reports of Web-based and Mobile Health Interventions
J Med Internet Res 2011;13(4):e126
URL: http://www.jmir.org/2011/4/e126/
doi: 10.2196/jmir.1923
PMID: 22209829

* Erforderlich

Your name *

First Last

David Daniel Ebert

Primary Affiliation (short), City, Country *

University of Toronto, Toronto, Canada

Department of Clinical, Neuro, and Developme

Your e-mail address *

abc@gmail.com

david.daniel.ebert@gmail.com

Title of your manuscript *

Provide the (draft) title of your manuscript.

Internet-based and mobile-supported stress management as a universal prevention approach – Effectiveness and moderators from a large pragmatic randomized controlled trial

Name of your App/Software/Intervention *

If there is a short and a long/alternate name, write the short name first and add the long name in brackets.

GET.ON Stress

Evaluated Version (if any)

e.g. "V1", "Release 2017-03-01", "Version 2.0.27913"

Meine Antwort

Language(s) *

What language is the intervention/app in? If multiple languages are available, separate by comma (e.g. "English, French")

German

URL of your Intervention Website or App

e.g. a direct link to the mobile app on app in appstore (itunes, Google Play), or URL of the website. If the intervention is a DVD or hardware, you can also link to an Amazon page.

Meine Antwort

URL of an image/screenshot (optional)

Meine Antwort

Accessibility *

Can an enduser access the intervention presently?

- ○ access is free and open
- ○ access only for special usergroups, not open
- ◉ access is open to everyone, but requires payment/subscription/in-app purchases
- ○ app/intervention no longer accessible
- ○ Sonstiges:

Primary Medical Indication/Disease/Condition *

e.g. "Stress", "Diabetes", or define the target group in brackets after the condition, e.g. "Autism (Parents of children with)", "Alzheimers (Informal Caregivers of)"

Stress

Primary Outcomes measured in trial *

comma-separated list of primary outcomes reported in the trial

perceived stress (PSS-10)

Secondary/other outcomes

Are there any other outcomes the intervention is expected to affect?

depression (CES-D), mental health related quality of life (AQoL-8), resilience (CDRISC), emotional exhaustion (MBI-EE), mental well-being (WEMWBS), psychological well-being (WHO-5), effort-reward imbalance (ERI-S), occupational self-efficacy (OSES), work engagement (UWES), productivity loss (WLQ-8), absenteeism and presentism days (TIC-P, 2 items), several moderators (personality, motivation for treatment, self-regulation competencies, general self-efficacy)

Recommended "Dose" *

What do the instructions for users say on how often the app should be used?

- ○ Approximately Daily
- ◉ Approximately Weekly
- ○ Approximately Monthly
- ○ Approximately Yearly
- ○ "as needed"
- ○ Sonstiges:

Approx. Percentage of Users (starters) still using the app as recommended after 3 months *

- (x) unknown / not evaluated
- ( ) 0-10%
- ( ) 11-20%
- ( ) 21-30%
- ( ) 31-40%
- ( ) 41-50%
- ( ) 51-60%
- ( ) 61-70%
- ( ) 71%-80%
- ( ) 81-90%
- ( ) 91-100%
- ( ) Sonstiges:

Overall, was the app/intervention effective? *

- (x) yes: all primary outcomes were significantly better in intervention group vs control
- ( ) partly: SOME primary outcomes were significantly better in intervention group vs control
- ( ) no statistically significant difference between control and intervention
- ( ) potentially harmful: control was significantly better than intervention in one or more outcomes
- ( ) inconclusive: more research is needed
- ( ) Sonstiges:

Article Preparation Status/Stage *

At which stage in your article preparation are you currently (at the time you fill in this form)

- ○ not submitted yet - in early draft status
- ○ not submitted yet - in late draft status, just before submission
- ○ submitted to a journal but not reviewed yet
- ◉ submitted to a journal and after receiving initial reviewer comments
- ○ submitted to a journal and accepted, but not published yet
- ○ published
- ○ Sonstiges:

Journal *

If you already know where you will submit this paper (or if it is already submitted), please provide the journal name (if it is not JMIR, provide the journal name under "other")

- ○ not submitted yet / unclear where I will submit this
- ◉ Journal of Medical Internet Research (JMIR)
- ○ JMIR mHealth and UHealth
- ○ JMIR Serious Games
- ○ JMIR Mental Health
- ○ JMIR Public Health
- ○ JMIR Formative Research
- ○ Other JMIR sister journal
- ○ Sonstiges:

Is this a full powered effectiveness trial or a pilot/feasibility trial? *

- ○ Pilot/feasibility
- ◉ Fully powered

Manuscript tracking number *

If this is a JMIR submission, please provide the manuscript tracking number under "other" (The ms tracking number can be found in the submission acknowledgement email, or when you login as author in JMIR. If the paper is already published in JMIR, then the ms tracking number is the four-digit number at the end of the DOI, to be found at the bottom of each published article in JMIR)

- ○ no ms number (yet) / not (yet) submitted to / published in JMIR
- ● Sonstiges: 22107

TITLE AND ABSTRACT

1a) TITLE: Identification as a randomized trial in the title

1a) Does your paper address CONSORT item 1a? *

I.e does the title contain the phrase "Randomized Controlled Trial"? (if not, explain the reason under "other")

- ● yes
- ○ Sonstiges:

1a-i) Identify the mode of delivery in the title

Identify the mode of delivery. Preferably use “web-based” and/or “mobile” and/or “electronic game” in the title. Avoid ambiguous terms like “online”, “virtual”, “interactive”. Use “Internet-based” only if Intervention includes non-web-based Internet components (e.g. email), use “computer-based” or “electronic” only if offline products are used. Use “virtual” only in the context of “virtual reality” (3-D worlds). Use “online” only in the context of “online support groups”. Complement or substitute product names with broader terms for the class of products (such as “mobile” or “smart phone” instead of “iphone”), especially if the application runs on different platforms.

	1	2	3	4	5	
subitem not at all important	○	○	○	○	●	essential

Auswahl löschen

Does your paper address subitem 1a-i? *

Copy and paste relevant sections from manuscript title (include quotes in quotation marks "like this" to indicate direct quotes from your manuscript), or elaborate on this item by providing additional information not in the ms, or briefly explain why the item is not applicable/relevant for your study

Yes. "Internet-based and mobile-supported"

1a-ii) Non-web-based components or important co-interventions in title

Mention non-web-based components or important co-interventions in title, if any (e.g., "with telephone support").

	1	2	3	4	5	
subitem not at all important	○	○	○	○	○	essential

Does your paper address subitem 1a-ii?

Copy and paste relevant sections from manuscript title (include quotes in quotation marks "like this" to indicate direct quotes from your manuscript), or elaborate on this item by providing additional information not in the ms, or briefly explain why the item is not applicable/relevant for your study

Not applicable (no non-web-based components in addition to 1a-i)

1a-iii) Primary condition or target group in the title

Mention primary condition or target group in the title, if any (e.g., "for children with Type I Diabetes")
Example: A Web-based and Mobile Intervention with Telephone Support for Children with Type I Diabetes: Randomized Controlled Trial

	1	2	3	4	5	
subitem not at all important	○	○	○	◉	○	essential

Auswahl löschen

Does your paper address subitem 1a-iii? *

Copy and paste relevant sections from manuscript title (include quotes in quotation marks "like this" to indicate direct quotes from your manuscript), or elaborate on this item by providing additional information not in the ms, or briefly explain why the item is not applicable/relevant for your study

Yes, but it's defined as "as a universal prevention approach" in title (defining that it was open to everyone)

1b) ABSTRACT: Structured summary of trial design, methods, results, and conclusions

NPT extension: Description of experimental treatment, comparator, care providers, centers, and blinding status.

1b-i) Key features/functionalities/components of the intervention and comparator in the METHODS section of the ABSTRACT

Mention key features/functionalities/components of the intervention and comparator in the abstract. If possible, also mention theories and principles used for designing the site. Keep in mind the needs of systematic reviewers and indexers by including important synonyms. (Note: Only report in the abstract what the main paper is reporting. If this information is missing from the main body of text, consider adding it)

	1	2	3	4	5	
subitem not at all important	○	○	○	○	◉	essential

Auswahl löschen

Does your paper address subitem 1b-i? *

Copy and paste relevant sections from the manuscript abstract (include quotes in quotation marks "like this" to indicate direct quotes from your manuscript), or elaborate on this item by providing additional information not in the ms, or briefly explain why the item is not applicable/relevant for your study

Yes. "The iSMI consisted of seven sessions and one booster session and offered no therapeutic guidance."

1b-ii) Level of human involvement in the METHODS section of the ABSTRACT

Clarify the level of human involvement in the abstract, e.g., use phrases like "fully automated" vs. "therapist/nurse/care provider/physician-assisted" (mention number and expertise of providers involved, if any). (Note: Only report in the abstract what the main paper is reporting. If this information is missing from the main body of text, consider adding it)

	1	2	3	4	5	
subitem not at all important	○	○	○	○	◉	essential

Auswahl löschen

Does your paper address subitem 1b-ii?

Copy and paste relevant sections from the manuscript abstract (include quotes in quotation marks "like this" to indicate direct quotes from your manuscript), or elaborate on this item by providing additional information not in the ms, or briefly explain why the item is not applicable/relevant for your study

Yes. "offered no therapeutic guidance"

1b-iii) Open vs. closed, web-based (self-assessment) vs. face-to-face assessments in the METHODS section of the ABSTRACT

Mention how participants were recruited (online vs. offline), e.g., from an open access website or from a clinic or a closed online user group (closed usergroup trial), and clarify if this was a purely web-based trial, or there were face-to-face components (as part of the intervention or for assessment). Clearly say if outcomes were self-assessed through questionnaires (as common in web-based trials). Note: In traditional offline trials, an open trial (open-label trial) is a type of clinical trial in which both the researchers and participants know which treatment is being administered. To avoid confusion, use "blinded" or "unblinded" to indicated the level of blinding instead of "open", as "open" in web-based trials usually refers to "open access" (i.e. participants can self-enrol). (Note: Only report in the abstract what the main paper is reporting. If this information is missing from the main body of text, consider adding it)

	1	2	3	4	5	
subitem not at all important	○	○	○	◉	○	essential

Auswahl löschen

Does your paper address subitem 1b-iii?

Copy and paste relevant sections from the manuscript abstract (include quotes in quotation marks "like this" to indicate direct quotes from your manuscript), or elaborate on this item by providing additional information not in the ms, or briefly explain why the item is not applicable/relevant for your study

Yes. "Self-report data were assessed at baseline, seven weeks, and at six months following randomization."

1b-iv) RESULTS section in abstract must contain use data

Report number of participants enrolled/assessed in each group, the use/uptake of the intervention (e.g., attrition/adherence metrics, use over time, number of logins etc.), in addition to primary/secondary outcomes. (Note: Only report in the abstract what the main paper is reporting. If this information is missing from the main body of text, consider adding it)

	1	2	3	4	5	
subitem not at all important	○	○	●	○	○	essential

Auswahl löschen

Does your paper address subitem 1b-iv?

Copy and paste relevant sections from the manuscript abstract (include quotes in quotation marks "like this" to indicate direct quotes from your manuscript), or elaborate on this item by providing additional information not in the ms, or briefly explain why the item is not applicable/relevant for your study

"396 employees were randomly assigned to the intervention condition (IC) or the six-month waiting list control condition (WLC)." - Since focus of the study was moderators, the moderator effects were reported instead of the uptake.

1b-v) CONCLUSIONS/DISCUSSION in abstract for negative trials

Conclusions/Discussions in abstract for negative trials: Discuss the primary outcome - if the trial is negative (primary outcome not changed), and the intervention was not used, discuss whether negative results are attributable to lack of uptake and discuss reasons. (Note: Only report in the abstract what the main paper is reporting. If this information is missing from the main body of text, consider adding it)

	1	2	3	4	5	
subitem not at all important	○	○	○	○	●	essential

Auswahl löschen

Does your paper address subitem 1b-v?

Copy and paste relevant sections from the manuscript abstract (include quotes in quotation marks "like this" to indicate direct quotes from your manuscript), or elaborate on this item by providing additional information not in the ms, or briefly explain why the item is not applicable/relevant for your study

Not negative, but results are doscussed. "This study indicates that iSMIs can be effective in a broad range of employees with no need for pre-selection to achieve substantial effects. The subgroups that might not profit all had extreme values on the respective measures and represented only a very small proportion of the investigated sample, indicating a broad applicability of GET.ON Stress."

INTRODUCTION

2a) In INTRODUCTION: Scientific background and explanation of rationale

2a-i) Problem and the type of system/solution

Describe the problem and the type of system/solution that is object of the study: intended as stand-alone intervention vs. incorporated in broader health care program? Intended for a particular patient population? Goals of the intervention, e.g., being more cost-effective to other interventions, replace or complement other solutions? (Note: Details about the intervention are provided in “Methods” under 5)

	1	2	3	4	5	
subitem not at all important	○	○	○	◉	○	essential

Auswahl löschen

Does your paper address subitem 2a-i? *

Copy and paste relevant sections from the manuscript (include quotes in quotation marks "like this" to indicate direct quotes from your manuscript), or elaborate on this item by providing additional information not in the ms, or briefly explain why the item is not applicable/relevant for your study

Yes. After a description on how traditional treatment studies work (restricted to specific group), it's described how the intervention is intended to be used in this study. Summary is:
"Therefore, the aim of the current study was to evaluate the effects of the Internet- and mobile supported self-help intervention GET.ON Stress as a universal prevention approach, without preselecting participants based on symptom severity. Moreover, in an exploratory approach, a broad range of a priori selected potential effect modifiers were tested in a sample adequately powered for moderator analyses."

2a-ii) Scientific background, rationale: What is known about the (type of) system

Scientific background, rationale: What is known about the (type of) system that is the object of the study (be sure to discuss the use of similar systems for other conditions/diagnoses, if appropiate), motivation for the study, i.e. what are the reasons for and what is the context for this specific study, from which stakeholder viewpoint is the study performed, potential impact of findings [2]. Briefly justify the choice of the comparator.

	1	2	3	4	5	
subitem not at all important	○	○	○	◉	○	essential

Auswahl löschen

Does your paper address subitem 2a-ii? *

Copy and paste relevant sections from the manuscript (include quotes in quotation marks "like this" to indicate direct quotes from your manuscript), or elaborate on this item by providing additional information not in the ms, or briefly explain why the item is not applicable/relevant for your study

Yes. In-depth description on what is known and what is missing. Examples below:
"Even for the so far best evaluated iSMI GET.ON Stress [13,19–25] evidence is based on trials in which participants have been preselected based on a high baseline score in perceived stress (Perceived Stress Scale [26,27], PSS-10 ≥ 22). Using a broad range of eligibility criteria, however, can limit the real-life applicability of the results [28] as in routine preventive occupational health care, iSMIs are usually offered to a wide range of participants. Therefore, it is crucial to investigate treatment effects without pre-selection in a universal prevention approach [29]."
"The second shortcoming relates to the lack of moderator analyses. Even though Internet-based intervention research is rapidly growing, empirical data on moderators of IMIs [33–39] and in particular Internet occupational health interventions is scarce [34]. Furthermore, randomized controlled trials are usually powered to detect effects on the primary outcome, and hence underpowered to reliably test moderator hypotheses [40]. With reported numbers needed to treat of 5.43 for unguided iSMIs (and 4.20 in the overall sample) [14], it is clear however, that a substantial part of participants do not profit yet from taking part in an intervention. "

2b) In INTRODUCTION: Specific objectives or hypotheses

Does your paper address CONSORT subitem 2b? *

Copy and paste relevant sections from the manuscript (include quotes in quotation marks "like this" to indicate direct quotes from your manuscript), or elaborate on this item by providing additional information not in the ms, or briefly explain why the item is not applicable/relevant for your study

Therefore, the aim of the current study was to evaluate the effects of the Internet- and mobile supported self-help intervention GET.ON Stress as a universal prevention approach, without preselecting participants based on symptom severity. Moreover, in an exploratory approach, a broad range of a priori selected potential effect modifiers were tested in a sample adequately powered for moderator analyses.

METHODS

3a) Description of trial design (such as parallel, factorial) including allocation ratio

Does your paper address CONSORT subitem 3a? *

Copy and paste relevant sections from the manuscript (include quotes in quotation marks "like this" to indicate direct quotes from your manuscript), or elaborate on this item by providing additional information not in the ms, or briefly explain why the item is not applicable/relevant for your study

Yes. "A two-armed randomized controlled trial (RCT) (N=396) was conducted comparing a self-guided iSMI (GET.ON Stress) and a wait-list control condition (WLC), with both conditions having full access to treatment as usual (TAU)."

3b) Important changes to methods after trial commencement (such as eligibility criteria), with reasons

Does your paper address CONSORT subitem 3b? *

Copy and paste relevant sections from the manuscript (include quotes in quotation marks "like this" to indicate direct quotes from your manuscript), or elaborate on this item by providing additional information not in the ms, or briefly explain why the item is not applicable/relevant for your study

There were no changes to methods after trial commencement

3b-i) Bug fixes, Downtimes, Content Changes

Bug fixes, Downtimes, Content Changes: ehealth systems are often dynamic systems. A description of changes to methods therefore also includes important changes made on the intervention or comparator during the trial (e.g., major bug fixes or changes in the functionality or content) (5-iii) and other “unexpected events” that may have influenced study design such as staff changes, system failures/downtimes, etc. [2].

	1	2	3	4	5	
subitem not at all important	(●)	( )	( )	( )	( )	essential

Auswahl löschen

Does your paper address subitem 3b-i?

Copy and paste relevant sections from the manuscript (include quotes in quotation marks "like this" to indicate direct quotes from your manuscript), or elaborate on this item by providing additional information not in the ms, or briefly explain why the item is not applicable/relevant for your study

There were no bug fixes, downtimes or content changes during the span of the study.

4a) Eligibility criteria for participants

Does your paper address CONSORT subitem 4a? *

Copy and paste relevant sections from the manuscript (include quotes in quotation marks "like this" to indicate direct quotes from your manuscript), or elaborate on this item by providing additional information not in the ms, or briefly explain why the item is not applicable/relevant for your study

Yes. "Inclusion and exclusion criteria were kept to a minimum to mimic routine conditionsas closely as possible. Participants were included who (1) were 18 years and older, (2) were currently employed, (3) had Internet access, and (4) provided a valid email address.
Individuals were only excluded if they were at risk of suicide (indicated by a score of 2 or higher on the Beck suicide item (Beck, Steer, & Brown, 1996) or if they self-reported to have been diagnosed with psychosis or dissociative symptoms. Besides that, there were no restraints for participants, for example concerning low or high stress, depression or other symptom severity criteria.

4a-i) Computer / Internet literacy

Computer / Internet literacy is often an implicit “de facto” eligibility criterion - this should be explicitly clarified.

	1	2	3	4	5	
subitem not at all important	○	○	○	◉	○	essential

Auswahl löschen

Does your paper address subitem 4a-i?

Copy and paste relevant sections from the manuscript (include quotes in quotation marks "like this" to indicate direct quotes from your manuscript), or elaborate on this item by providing additional information not in the ms, or briefly explain why the item is not applicable/relevant for your study

The need for an Internet access and the possibility of providing a valid email adress are part of the eligibility criteria.

4a-ii) Open vs. closed, web-based vs. face-to-face assessments:

Open vs. closed, web-based vs. face-to-face assessments: Mention how participants were recruited (online vs. offline), e.g., from an open access website or from a clinic, and clarify if this was a purely web-based trial, or there were face-to-face components (as part of the intervention or for assessment), i.e., to what degree got the study team to know the participant. In online-only trials, clarify if participants were quasi-anonymous and whether having multiple identities was possible or whether technical or logistical measures (e.g., cookies, email confirmation, phone calls) were used to detect/prevent these.

	1	2	3	4	5	
subitem not at all important	○	○	○	○	◉	essential

Auswahl löschen

Does your paper address subitem 4a-ii? *

Copy and paste relevant sections from the manuscript (include quotes in quotation marks "like this" to indicate direct quotes from your manuscript), or elaborate on this item by providing additional information not in the ms, or briefly explain why the item is not applicable/relevant for your study

Yes. "Inclusion and exclusion criteria were kept to a minimum to mimic routine conditions as closely as possible. Participants were included who (1) were 18 years and older, (2) were currently employed, (3) had Internet access, and (4) provided a valid email address.
Individuals were only excluded if they were at risk of suicide (indicated by a score of 2 or higher on the Beck suicide item (Beck, Steer, & Brown, 1996)
or if they self-reported to have been diagnosed with psychosis or dissociative symptoms.
Besides that, there were no restraints for participants, for example concerning low or high stress, depression or other symptom severity criteria.

4a-iii) Information giving during recruitment

Information given during recruitment. Specify how participants were briefed for recruitment and in the informed consent procedures (e.g., publish the informed consent documentation as appendix, see also item X26), as this information may have an effect on user self-selection, user expectation and may also bias results.

	1	2	3	4	5	
subitem not at all important	○	○	◉	○	○	essential

Auswahl löschen

Does your paper address subitem 4a-iii?

Copy and paste relevant sections from the manuscript (include quotes in quotation marks "like this" to indicate direct quotes from your manuscript), or elaborate on this item by providing additional information not in the ms, or briefly explain why the item is not applicable/relevant for your study

"Interested individuals received an e-mail with detailed information about the study procedures and an invitation to take part in the study. If they were interested, they were asked to complete an online screening questionnaire. Individuals meeting the eligibility criteria completed the baseline assessment and were invited to complete the informed consent form. Once the full written informed consent was received, participants entered the study and were randomly allocated to one of the two study conditions. "

4b) Settings and locations where the data were collected

Does your paper address CONSORT subitem 4b? *

Copy and paste relevant sections from the manuscript (include quotes in quotation marks "like this" to indicate direct quotes from your manuscript), or elaborate on this item by providing additional information not in the ms, or briefly explain why the item is not applicable/relevant for your study

"The assessment took place on a secured online-based system with 256-bit AES encryption."

4b-i) Report if outcomes were (self-)assessed through online questionnaires

Clearly report if outcomes were (self-)assessed through online questionnaires (as common in web-based trials) or otherwise.

	1	2	3	4	5	
subitem not at all important	○	○	○	◉	○	essential

Auswahl löschen

Does your paper address subitem 4b-i? *

Copy and paste relevant sections from the manuscript (include quotes in quotation marks "like this" to indicate direct quotes from your manuscript), or elaborate on this item by providing additional information not in the ms, or briefly explain why the item is not applicable/relevant for your study

"All measures were assessed through online self-report questionnaires and are described below."

4b-ii) Report how institutional affiliations are displayed

Report how institutional affiliations are displayed to potential participants [on ehealth media], as affiliations with prestigious hospitals or universities may affect volunteer rates, use, and reactions with regards to an intervention.(Not a required item – describe only if this may bias results)

	1	2	3	4	5	
subitem not at all important	○	○	◉	○	○	essential

Auswahl löschen

Does your paper address subitem 4b-ii?

Copy and paste relevant sections from the manuscript (include quotes in quotation marks "like this" to indicate direct quotes from your manuscript), or elaborate on this item by providing additional information not in the ms, or briefly explain why the item is not applicable/relevant for your study

Yes, it was specified that recruitment was affiliated with a large health insurance company "Participants were recruited via the occupational health program of a large health insurance company in Germany (BARMER)"

5) The interventions for each group with sufficient details to allow replication, including how and when they were actually administered

5-i) Mention names, credential, affiliations of the developers, sponsors, and owners

Mention names, credential, affiliations of the developers, sponsors, and owners [6] (if authors/evaluators are owners or developer of the software, this needs to be declared in a "Conflict of interest" section or mentioned elsewhere in the manuscript).

	1	2	3	4	5	
subitem not at all important	○	○	◉	○	○	essential

Auswahl löschen

Does your paper address subitem 5-i?

Copy and paste relevant sections from the manuscript (include quotes in quotation marks "like this" to indicate direct quotes from your manuscript), or elaborate on this item by providing additional information not in the ms, or briefly explain why the item is not applicable/relevant for your study

It was used a already developed interventions that was used in other studies with a more narrow population. The original publications with their authors being the creators are clearly referenced.
"iSMI GET.ON Stress [13,19–25] "

5-ii) Describe the history/development process

Describe the history/development process of the application and previous formative evaluations (e.g., focus groups, usability testing), as these will have an impact on adoption/use rates and help with interpreting results.

	1	2	3	4	5	
subitem not at all important	○	○	◉	○	○	essential

Auswahl löschen

Does your paper address subitem 5-ii?

Copy and paste relevant sections from the manuscript (include quotes in quotation marks "like this" to indicate direct quotes from your manuscript), or elaborate on this item by providing additional information not in the ms, or briefly explain why the item is not applicable/relevant for your study

As described below, the intervention was already used in other studies that are referenced:
"iSMI GET.ON Stress [13,19–25]"

5-iii) Revisions and updating

Revisions and updating. Clearly mention the date and/or version number of the application/intervention (and comparator, if applicable) evaluated, or describe whether the intervention underwent major changes during the evaluation process, or whether the development and/or content was "frozen" during the trial. Describe dynamic components such as news feeds or changing content which may have an impact on the replicability of the intervention (for unexpected events see item 3b).

	1	2	3	4	5	
subitem not at all important	○	◉	○	○	○	essential

Auswahl löschen

Does your paper address subitem 5-iii?

Copy and paste relevant sections from the manuscript (include quotes in quotation marks "like this" to indicate direct quotes from your manuscript), or elaborate on this item by providing additional information not in the ms, or briefly explain why the item is not applicable/relevant for your study

The training is described in much detail with all parts. The description makes it clear that the intervention was a complete un-changed package.

5-iv) Quality assurance methods

Provide information on quality assurance methods to ensure accuracy and quality of information provided [1], if applicable.

	1	2	3	4	5	
subitem not at all important	○	○	◉	○	○	essential

Auswahl löschen

Does your paper address subitem 5-iv?

Copy and paste relevant sections from the manuscript (include quotes in quotation marks "like this" to indicate direct quotes from your manuscript), or elaborate on this item by providing additional information not in the ms, or briefly explain why the item is not applicable/relevant for your study

Quality assurance was done by using an intervention that was proven effective in other studies (and it was also rooted in a well-known stress model)

5-v) Ensure replicability by publishing the source code, and/or providing screenshots/screen-capture video, and/or providing flowcharts of the algorithms used

Ensure replicability by publishing the source code, and/or providing screenshots/screen-capture video, and/or providing flowcharts of the algorithms used. Replicability (i.e., other researchers should in principle be able to replicate the study) is a hallmark of scientific reporting.

	1	2	3	4	5	
subitem not at all important	○	○	◉	○	○	essential

Auswahl löschen

Does your paper address subitem 5-v?

Copy and paste relevant sections from the manuscript (include quotes in quotation marks "like this" to indicate direct quotes from your manuscript), or elaborate on this item by providing additional information not in the ms, or briefly explain why the item is not applicable/relevant for your study

Source code or screen-capture could not be provided since the intervention is commercially available. The intervention content and all the different components are described in great detail (in this paper as well as in the referenced original papers), however, which would allow for conceptual replicability (but not technically).

5-vi) Digital preservation

Digital preservation: Provide the URL of the application, but as the intervention is likely to change or disappear over the course of the years; also make sure the intervention is archived (Internet Archive, webcitation.org, and/or publishing the source code or screenshots/videos alongside the article). As pages behind login screens cannot be archived, consider creating demo pages which are accessible without login.

	1	2	3	4	5	
subitem not at all important	○	○	◉	○	○	essential

Auswahl löschen

Does your paper address subitem 5-vi?

Copy and paste relevant sections from the manuscript (include quotes in quotation marks "like this" to indicate direct quotes from your manuscript), or elaborate on this item by providing additional information not in the ms, or briefly explain why the item is not applicable/relevant for your study

See answer before.

5-vii) Access

Access: Describe how participants accessed the application, in what setting/context, if they had to pay (or were paid) or not, whether they had to be a member of specific group. If known, describe how participants obtained “access to the platform and Internet” [1]. To ensure access for editors/reviewers/readers, consider to provide a “backdoor” login account or demo mode for reviewers/readers to explore the application (also important for archiving purposes, see vi).

	1	2	3	4	5	
subitem not at all important	○	○	○	○	○	essential

Does your paper address subitem 5-vii? *

Copy and paste relevant sections from the manuscript (include quotes in quotation marks "like this" to indicate direct quotes from your manuscript), or elaborate on this item by providing additional information not in the ms, or briefly explain why the item is not applicable/relevant for your study

Access was provided for everyone interested. It was however, most certainly shaped by the recruitment:
"Participants were recruited via the occupational health program of a large health insurance company in Germany (BARMER) in a way that mimics the intended implementation of the intervention in routine practice in the future, i.e., by offering the intervention in a public occupational mental health approach to the general working population in an online setting."
Moreover, participantes needed to complete baseline assesment and give written informed consent before they had access to the intervention (when randomized to intervention group)
"Interested individuals received an e-mail with detailed information about the study procedures, were asked to provide an e-mail address and to complete an online screening questionnaire. Individuals meeting the eligibility criteria completed the baseline assessment and were invited to complete the informed consent form. Once the full written informed consent was received, participants entered the study and were randomly allocated to one of the two study conditions."

5-viii) Mode of delivery, features/functionalities/components of the intervention and comparator, and the theoretical framework

Describe mode of delivery, features/functionalities/components of the intervention and comparator, and the theoretical framework [6] used to design them (instructional strategy [1], behaviour change techniques, persuasive features, etc., see e.g., [7, 8] for terminology). This includes an in-depth description of the content (including where it is coming from and who developed it) [1]," whether [and how] it is tailored to individual circumstances and allows users to track their progress and receive feedback" [6]. This also includes a description of communication delivery channels and – if computer-mediated communication is a component – whether communication was synchronous or asynchronous [6]. It also includes information on presentation strategies [1], including page design principles, average amount of text on pages, presence of hyperlinks to other resources, etc. [1].

	1	2	3	4	5	
subitem not at all important	○	○	○	○	◉	essential

Auswahl löschen

Does your paper address subitem 5-viii? *

Copy and paste relevant sections from the manuscript (include quotes in quotation marks "like this" to indicate direct quotes from your manuscript), or elaborate on this item by providing additional information not in the ms, or briefly explain why the item is not applicable/relevant for your study

Yes. This information is provided in the "Intervention" section in the methods.

"As this study evaluates an intervention that was evaluated in an indicated prevention context before, intervention content and delivery methods mirror those in related studies [20].
The training GET.ON Stress is based on Lazarus' transactional model of stress [43]. According to this model, there are two coping strategies to deal with stress. On the one side, there can be a problem-oriented approach in changing the situation. The other approach focusses on situations where only the evaluation of the situation can be adjusted; this is called emotional regulation. Therefore, the training focuses on strategies for systematic problem-solving as well as emotion-regulation.
The intervention consists of seven regular sessions and one booster session (provided four weeks after the last regular session was completed). The program starts with psychoeducation about stress (session 1) which guides participants to understand and find coping strategies for common problem situations. Based on their personal stressors, participants are asked to record their goals and motivation for the training. This is followed by two sessions focusing on problem-solving skills (sessions 2-3) where participants are guided through a six-step method based on problem-solving therapy [44]. The personal plan shall be implemented between the sessions before participants are asked to redo the exercise with the same or a different problem. The next part of the training (sessions 4-6) is based on the Affect Regulation training [45,46]. Each session focusses on one emotion regulation strategy: muscle and breathing relaxation, acceptance of emotions as well as effective self-support with 15-minute audio files guiding users through the different exercises. Participants are encouraged to redo the exercises between sessions on a daily basis. The regular training is completed by a session guiding the creation of a "Plan for the future" (session 7), in which users are asked to review their progress and develop strategies according to their personal stress indicators in the future. Moreover, they are guided to write a letter to their future self. The booster session reviews the essential content of the intervention and allows participants to reassess their goals and plans for the future. Additionally, participants could choose from an array of elective modules in sessions 2 to 6 addressing common stress-related topics such as time management, sleep hygiene, or social support. Participants were advised to complete 1-2 sessions per week.
Across sessions, the training was composed of interactive education, audio and video files, and additional downloadable material. Additionally, testimonials offered impulses and examples concerning the exercises. The intervention is based on responsive design, meaning that it could be completed on a desktop computer, tablet or smartphone. Through providing multiple choices among various response options, the content of the intervention was automatically tailored to the specific

needs and interests of the individual participants. To integrate the newly acquired knowledge into daily life, homework assignments and behavioral planning were a crucial part of the training. Online diaries were also offered. The training was not accompanied by therapeutic guidance. However, the participants could opt-in for automatic text messages on their mobile phones choosing either a light (1 text message every other day) or intensive support (2-3 text messages per day). Text messages focused on ultra-brief exercises to be carried out in daily life routine, aiming to facilitate transfer from training into real life."

5-ix) Describe use parameters

Describe use parameters (e.g., intended “doses” and optimal timing for use). Clarify what instructions or recommendations were given to the user, e.g., regarding timing, frequency, heaviness of use, if any, or was the intervention used ad libitum.

	1	2	3	4	5	
subitem not at all important	○	○	○	◉	○	essential

Auswahl löschen

Does your paper address subitem 5-ix?

Copy and paste relevant sections from the manuscript (include quotes in quotation marks "like this" to indicate direct quotes from your manuscript), or elaborate on this item by providing additional information not in the ms, or briefly explain why the item is not applicable/relevant for your study

"Participants were advised to complete 1-2 sessions per week."

5-x) Clarify the level of human involvement

Clarify the level of human involvement (care providers or health professionals, also technical assistance) in the e-intervention or as co-intervention (detail number and expertise of professionals involved, if any, as well as “type of assistance offered, the timing and frequency of the support, how it is initiated, and the medium by which the assistance is delivered”. It may be necessary to distinguish between the level of human involvement required for the trial, and the level of human involvement required for a routine application outside of a RCT setting (discuss under item 21 – generalizability).

	1	2	3	4	5	
subitem not at all important	○	○	○	○	○	essential

Does your paper address subitem 5-x?

Copy and paste relevant sections from the manuscript (include quotes in quotation marks "like this" to indicate direct quotes from your manuscript), or elaborate on this item by providing additional information not in the ms, or briefly explain why the item is not applicable/relevant for your study

"The training was not accompanied by therapeutic guidance. However, the participants could opt-in for automatic text messages on their mobile phones choosing either a light (1 text message every other day) or intensive support (2-3 text messages per day)."

5-xi) Report any prompts/reminders used

Report any prompts/reminders used: Clarify if there were prompts (letters, emails, phone calls, SMS) to use the application, what triggered them, frequency etc. It may be necessary to distinguish between the level of prompts/reminders required for the trial, and the level of prompts/reminders for a routine application outside of a RCT setting (discuss under item 21 – generalizability).

	1	2	3	4	5	
subitem not at all important	○	○	○	◉	○	essential

Auswahl löschen

Does your paper address subitem 5-xi? *

Copy and paste relevant sections from the manuscript (include quotes in quotation marks "like this" to indicate direct quotes from your manuscript), or elaborate on this item by providing additional information not in the ms, or briefly explain why the item is not applicable/relevant for your study

Same as before "The training was not accompanied by therapeutic guidance. However, the participants could opt-in for automatic text messages on their mobile phones choosing either a light (1 text message every other day) or intensive support (2-3 text messages per day)."

5-xii) Describe any co-interventions (incl. training/support)

Describe any co-interventions (incl. training/support): Clearly state any interventions that are provided in addition to the targeted eHealth intervention, as ehealth intervention may not be designed as stand-alone intervention. This includes training sessions and support [1]. It may be necessary to distinguish between the level of training required for the trial, and the level of training for a routine application outside of a RCT setting (discuss under item 21 – generalizability.

	1	2	3	4	5	
subitem not at all important	○	○	◉	○	○	essential

Auswahl löschen

Does your paper address subitem 5-xii? *

Copy and paste relevant sections from the manuscript (include quotes in quotation marks "like this" to indicate direct quotes from your manuscript), or elaborate on this item by providing additional information not in the ms, or briefly explain why the item is not applicable/relevant for your study

There were no co-interventions.

6a) Completely defined pre-specified primary and secondary outcome measures, including how and when they were assessed

Does your paper address CONSORT subitem 6a? *

Copy and paste relevant sections from the manuscript (include quotes in quotation marks "like this" to indicate direct quotes from your manuscript), or elaborate on this item by providing additional information not in the ms, or briefly explain why the item is not applicable/relevant for your study

Yes.
"The primary outcome was the perceived level of stress as measured by the German version of the PSS-10 [26,27] since it is based on Lazarus' transactional model of stress. The 10 items were answered on a 5-point Likert scale (ranging from 0=never to 4=very often) referring to the past week. Due to its sum score, total scale scores range from 0 to 40. Cronbach's alpha was .83 at T1, .91 at T2 and .91 at T3 in this study.
Secondary outcomes included measurement of mental health and work-related outcomes. Among those concerning mental health, the following outcomes were measured using the specified scales: depression, using the German adaptation of the Center for Epidemiological Studies' Depression Scale (CES-D; 15 items; range 0-45; α=.94) [47,48]; Mental health related quality of life, Assessment of Quality of Life (AQoL; 8 items; range 8-41; α=.84) [49]; resilience, using the Connor-Davidson Resilience Scale (CD-RISC; 10 items; range 10-50; α=.93) [50]; emotional exhaustion, using the subscale of the Maslach-Burnout-Inventory (MBI-EE; 5 items; range 1-6; α=.93) [51]; mental well-being, using the Warwick-Edinburgh Mental Wellbeing Scale (WEMWBS; 14 items; range 14-84; α=.96) [52]; psychological well-being, using the 5-item World-Health-Organization Well-Being-Index (WHO-5; 5 items; range 0-100; α=.91) [53]. The following work-related outcomes were assessed: effort-reward imbalance, using the Effort-Reward-Imbalance Questionnaire – Short Form (ERI-S; 10 items; α=.84) [54]; occupational self-efficacy, using the Occupational Self-Efficacy Scale (OSES; 6 items; range 6-36; α=.93) [55]; work engagement, using the Utrecht Work-Engagement-Scale (UWES; 9 items; range 0-6; α=.97) [56]; productivity loss, using the Work-Limitations-Questionnaire (WLQ-8; 8 items; α=.87) [57]; absenteeism and presentism days, using the respective questions from the German version of the Trimbos and Institute of Medical Technology Assessment Cost Questionnaire for Psychiatry (TIC-P; 2 items) [58].
In addition, the following variables were assessed to be tested as potential moderators: Personality, using the Big-Five-Inventory (BFI-10; 10 items; subscale range α=.20-.77) [59]; Motivation for treatment, using the Psychotherapy Motivation Questionnaire – Short Form (FPTM; 4 items; α=.36) [60]; self-regulation competencies, using the Self-Regulation scale (SRS; 10 items; range 10-40; α=.84) [61]; general self-efficacy, using the General Self-Efficacy Scale (SWE; 10 items; range 10-40; α=.90) [62]."

6a-i) Online questionnaires: describe if they were validated for online use and apply CHERRIES items to describe how the questionnaires were designed/deployed

If outcomes were obtained through online questionnaires, describe if they were validated for online use and apply CHERRIES items to describe how the questionnaires were designed/deployed [9].

	1	2	3	4	5	
subitem not at all important	○	○	○	○	○	essential

Does your paper address subitem 6a-i?

Copy and paste relevant sections from manuscript text

Meine Antwort

6a-ii) Describe whether and how “use” (including intensity of use/dosage) was defined/measured/monitored

Describe whether and how “use” (including intensity of use/dosage) was defined/measured/monitored (logins, logfile analysis, etc.). Use/adoption metrics are important process outcomes that should be reported in any ehealth trial.

	1	2	3	4	5	
subitem not at all important	○	○	○	●	○	essential

Auswahl löschen

Does your paper address subitem 6a-ii?

Copy and paste relevant sections from manuscript text

Use was discussed under "Adherence" and was measured by session completion "Of the 198 participants in the IG, 188 (95.0%) finished session 1. Session 2 was completed by 171 (86.4%), session 3 by 154 (77.8%), session 4 by 135 (68.2%), session 5 by 121 (61.1%), session 6 by 108 (54.5%), session 7 by 94 (47.5%) and the booster session by 66 (33.3%) individuals. On average, participants worked through 5.23 (SD=2.74) sessions representing 65.5% of the intervention.

6a-iii) Describe whether, how, and when qualitative feedback from participants was obtained

Describe whether, how, and when qualitative feedback from participants was obtained (e.g., through emails, feedback forms, interviews, focus groups).

	1	2	3	4	5	
subitem not at all important	○	○	◉	○	○	essential

Auswahl löschen

Does your paper address subitem 6a-iii?

Copy and paste relevant sections from manuscript text

It was not obtained as it was not the focus of the study.

6b) Any changes to trial outcomes after the trial commenced, with reasons

Does your paper address CONSORT subitem 6b? *

Copy and paste relevant sections from the manuscript (include quotes in quotation marks "like this" to indicate direct quotes from your manuscript), or elaborate on this item by providing additional information not in the ms, or briefly explain why the item is not applicable/relevant for your study

No, since there were no changes to trial outcomes after trial commenced.

7a) How sample size was determined

NPT: When applicable, details of whether and how the clustering by care provides or centers was addressed

7a-i) Describe whether and how expected attrition was taken into account when calculating the sample size

Describe whether and how expected attrition was taken into account when calculating the sample size.

	1	2	3	4	5	
subitem not at all important	○	○	◉	○	○	essential

Auswahl löschen

Does your paper address subitem 7a-i?

Copy and paste relevant sections from manuscript title (include quotes in quotation marks "like this" to indicate direct quotes from your manuscript), or elaborate on this item by providing additional information not in the ms, or briefly explain why the item is not applicable/relevant for your study

Yes. However, not attrition but power needed for detecting moderator effects were taken into account.
"The sample size of the study was optimized to detect potential moderator effects. Previous studies on the same intervention found effects on the primary outcome perceived stress at T2 ranging from d = .84 to d = .93 [13,23,63] in highly stressed employees. Assuming potentially lower effects, due to the inclusion of less impaired employees, of approximately d = .60, we would have needed to include n=90 participants. Given that simulation studies showed that the sample size required to detect an interaction effect of the same magnitude needs to be at least three to fourfold, compare to the sample sizes needed for the primary analyses [64], we included N = 396 participants. This sample allowed us not only to detect a small regression effect of f^2 with a power (1-ß) of 80% and an error probability (α) of 0.05 but also a small intergroup effect size of d = .25 in a one-tailed test (calculated with G*Power)."

7b) When applicable, explanation of any interim analyses and stopping guidelines

Does your paper address CONSORT subitem 7b? *

Copy and paste relevant sections from the manuscript (include quotes in quotation marks "like this" to indicate direct quotes from your manuscript), or elaborate on this item by providing additional information not in the ms, or briefly explain why the item is not applicable/relevant for your study

There were no interim analyses or stopping guidelines due to the low-threshold nature of the intervention and experience of previous studies with the same intervention.

8a) Method used to generate the random allocation sequence

NPT: When applicable, how care providers were allocated to each trial group

Does your paper address CONSORT subitem 8a? *

Copy and paste relevant sections from the manuscript (include quotes in quotation marks "like this" to indicate direct quotes from your manuscript), or elaborate on this item by providing additional information not in the ms, or briefly explain why the item is not applicable/relevant for your study

Yes.
"Randomization took place at a ratio of 1:1. An independent third party who did not have any information about the participants performed the allocation. Randomization was carried out using an automated computer-based random integer generator (randlist). Other researchers could not bias the randomization process since participants were randomized in order of the incoming informed consent form."

8b) Type of randomisation; details of any restriction (such as blocking and block size)

Does your paper address CONSORT subitem 8b? *

Copy and paste relevant sections from the manuscript (include quotes in quotation marks "like this" to indicate direct quotes from your manuscript), or elaborate on this item by providing additional information not in the ms, or briefly explain why the item is not applicable/relevant for your study

Yes. "Randomization took place at a ratio of 1:1."

9) Mechanism used to implement the random allocation sequence (such as sequentially numbered containers), describing any steps taken to conceal the sequence until interventions were assigned

Does your paper address CONSORT subitem 9? *

Copy and paste relevant sections from the manuscript (include quotes in quotation marks "like this" to indicate direct quotes from your manuscript), or elaborate on this item by providing additional information not in the ms, or briefly explain why the item is not applicable/relevant for your study

Yes. "Randomization was carried out using an automated computer-based random integer generator (randlist). Other researchers could not bias the randomization process since participants were randomized in order of the incoming informed consent form. Participants were not blinded to study conditions. During the randomization process, the allocation was concealed from participants, researchers involved in recruitment, and study administration team."

10) Who generated the random allocation sequence, who enrolled participants, and who assigned participants to interventions

Does your paper address CONSORT subitem 10? *

Copy and paste relevant sections from the manuscript (include quotes in quotation marks "like this" to indicate direct quotes from your manuscript), or elaborate on this item by providing additional information not in the ms, or briefly explain why the item is not applicable/relevant for your study

Yes.
"Once the full written informed consent was received, participants entered the study and were randomly allocated to one of the two study conditions."
"An independent third party who did not have any information about the participants performed the allocation."

11a) If done, who was blinded after assignment to interventions (for example, participants, care providers, those assessing outcomes) and how

NPT: Whether or not administering co-interventions were blinded to group assignment

11a-i) Specify who was blinded, and who wasn't

Specify who was blinded, and who wasn't. Usually, in web-based trials it is not possible to blind the participants [1, 3] (this should be clearly acknowledged), but it may be possible to blind outcome assessors, those doing data analysis or those administering co-interventions (if any).

	1	2	3	4	5	
subitem not at all important	○	○	◉	○	○	essential

Auswahl löschen

Does your paper address subitem 11a-i? *

Copy and paste relevant sections from the manuscript (include quotes in quotation marks "like this" to indicate direct quotes from your manuscript), or elaborate on this item by providing additional information not in the ms, or briefly explain why the item is not applicable/relevant for your study

Yes.
"Participants were not blinded to study conditions. During the randomization process, the allocation was concealed from participants, researchers involved in recruitment, and study administration team."

11a-ii) Discuss e.g., whether participants knew which intervention was the "intervention of interest" and which one was the "comparator"

Informed consent procedures (4a-ii) can create biases and certain expectations - discuss e.g., whether participants knew which intervention was the "intervention of interest" and which one was the "comparator".

	1	2	3	4	5	
subitem not at all important	○	○	○	◉	○	essential

Auswahl löschen

Does your paper address subitem 11a-ii?

Copy and paste relevant sections from the manuscript (include quotes in quotation marks "like this" to indicate direct quotes from your manuscript), or elaborate on this item by providing additional information not in the ms, or briefly explain why the item is not applicable/relevant for your study

Yes. Due to the type of comparator (wait-list control) it was obvious which group was the intervention of interest.
"Participants were not blinded to study conditions."

11b) If relevant, description of the similarity of interventions

(this item is usually not relevant for ehealth trials as it refers to similarity of a placebo or sham intervention to a active medication/intervention)

Does your paper address CONSORT subitem 11b? *

Copy and paste relevant sections from the manuscript (include quotes in quotation marks "like this" to indicate direct quotes from your manuscript), or elaborate on this item by providing additional information not in the ms, or briefly explain why the item is not applicable/relevant for your study

No, not applicable since the control group was not active.

12a) Statistical methods used to compare groups for primary and secondary outcomes

NPT: When applicable, details of whether and how the clustering by care providers or centers was addressed

Does your paper address CONSORT subitem 12a? *

Copy and paste relevant sections from the manuscript (include quotes in quotation marks "like this" to indicate direct quotes from your manuscript), or elaborate on this item by providing additional information not in the ms, or briefly explain why the item is not applicable/relevant for your study

Yes.
"Outcome levels of IG and WLC were compared at T2 and T3 using analysis of covariance (ANCOVA) with baseline levels as covariates. Additionally, Cohen's d with 95% confidence intervals (CIs) were calculated based on the imputed dataset by comparing means and standard deviations (SDs) of the two groups at the respective time points. Furthermore, the difference in means along with its 95% CI was calculated. To improve interpretability, reliable change was calculated according to the method of Jacobson and Truax [67], as an indicator of the number of participants with treatment response."

12a-i) Imputation techniques to deal with attrition / missing values

Imputation techniques to deal with attrition / missing values: Not all participants will use the intervention/comparator as intended and attrition is typically high in ehealth trials. Specify how participants who did not use the application or dropped out from the trial were treated in the statistical analysis (a complete case analysis is strongly discouraged, and simple imputation techniques such as LOCF may also be problematic [4]).

	1	2	3	4	5	
subitem not at all important	○	○	○	○	◉	essential

Auswahl löschen

Does your paper address subitem 12a-i? *

Copy and paste relevant sections from the manuscript (include quotes in quotation marks "like this" to indicate direct quotes from your manuscript), or elaborate on this item by providing additional information not in the ms, or briefly explain why the item is not applicable/relevant for your study

Yes.
"following the intention-to-treat principle (ITT) missing data was handled with multiple imputations [66], using 100 estimations per missing value."

12b) Methods for additional analyses, such as subgroup analyses and adjusted analyses

Does your paper address CONSORT subitem 12b? *

Copy and paste relevant sections from the manuscript (include quotes in quotation marks "like this" to indicate direct quotes from your manuscript), or elaborate on this item by providing additional information not in the ms, or briefly explain why the item is not applicable/relevant for your study

Yes.
"Regression analyses were used to test baseline moderator X intervention condition interaction effects, using the SPSS macro PROCESS [62]. In case of a significant interaction effect, we applied the Johnson-Neyman-Technique [62,63] which tests the conditional effect of X on Y at different values along the continuum of the moderator and calculates transition points where the effect changes between statistically significant and not significant (at the α level of significance)."

X26) REB/IRB Approval and Ethical Considerations [recommended as subheading under "Methods"] (not a CONSORT item)

X26-i) Comment on ethics committee approval

	1	2	3	4	5	
subitem not at all important	○	◉	○	○	○	essential

Auswahl löschen

Does your paper address subitem X26-i?

Copy and paste relevant sections from the manuscript (include quotes in quotation marks "like this" to indicate direct quotes from your manuscript), or elaborate on this item by providing additional information not in the ms, or briefly explain why the item is not applicable/relevant for your study

No. Since the study was a secondary analysis of the main paper (which is referenced in the manuscript), the Ethics approval is not stated once again.

x26-ii) Outline informed consent procedures

Outline informed consent procedures e.g., if consent was obtained offline or online (how? Checkbox, etc.?), and what information was provided (see 4a-ii). See [6] for some items to be included in informed consent documents.

	1	2	3	4	5	
subitem not at all important	○	○	○	○	○	essential

Does your paper address subitem X26-ii?

Copy and paste relevant sections from the manuscript (include quotes in quotation marks "like this" to indicate direct quotes from your manuscript), or elaborate on this item by providing additional information not in the ms, or briefly explain why the item is not applicable/relevant for your study

Meine Antwort

X26-iii) Safety and security procedures

Safety and security procedures, incl. privacy considerations, and any steps taken to reduce the likelihood or detection of harm (e.g., education and training, availability of a hotline)

	1	2	3	4	5	
subitem not at all important	O	O	O	O	O	essential

Does your paper address subitem X26-iii?

Copy and paste relevant sections from the manuscript (include quotes in quotation marks "like this" to indicate direct quotes from your manuscript), or elaborate on this item by providing additional information not in the ms, or briefly explain why the item is not applicable/relevant for your study

Meine Antwort

RESULTS

13a) For each group, the numbers of participants who were randomly assigned, received intended treatment, and were analysed for the primary outcome

NPT: The number of care providers or centers performing the intervention in each group and the number of patients treated by each care provider in each center

Does your paper address CONSORT subitem 13a? *

Copy and paste relevant sections from the manuscript (include quotes in quotation marks "like this" to indicate direct quotes from your manuscript), or elaborate on this item by providing additional information not in the ms, or briefly explain why the item is not applicable/relevant for your study

Yes. Detailed information is depicted in Figure 1 and Table 1.
"The study flow is illustrated in Figure 1. All N = 396 participants provided data at T1. At T2 (T3) 176/396 or 88.8% (134/396; 67.7%) participants in the IG and 187/396 or 94.4% (179/396; 90.4%) in the WLC group completed the assessment."

13b) For each group, losses and exclusions after randomisation, together with reasons

Does your paper address CONSORT subitem 13b? (NOTE: Preferably, this is shown in a CONSORT flow diagram) *

Copy and paste relevant sections from the manuscript (include quotes in quotation marks "like this" to indicate direct quotes from your manuscript), or elaborate on this item by providing additional information not in the ms, or briefly explain why the item is not applicable/relevant for your study

Yes (but there was no loss at baseline due to randomization being done after the baseline questionnaire). Also depicted in CONSORTT flow diagram.
"The study flow is illustrated in Figure 1. All N = 396 participants provided data at T1. At T2 (T3) 176/396 or 88.8% (134/396; 67.7%) participants in the IG and 187/396 or 94.4% (179/396; 90.4%) in the WLC group completed the assessment."

13b-i) Attrition diagram

Strongly recommended: An attrition diagram (e.g., proportion of participants still logging in or using the intervention/comparator in each group plotted over time, similar to a survival curve) or other figures or tables demonstrating usage/dose/engagement.

	1	2	3	4	5	
subitem not at all important	○	◉	○	○	○	essential

Auswahl löschen

Does your paper address subitem 13b-i?

Copy and paste relevant sections from the manuscript or cite the figure number if applicable (include quotes in quotation marks "like this" to indicate direct quotes from your manuscript), or elaborate on this item by providing additional information not in the ms, or briefly explain why the item is not applicable/relevant for your study

No. Due to the time frame of the study and the focus on moderation effects, longterm
use of the intervention was not monitored.

14a) Dates defining the periods of recruitment and follow-up

Does your paper address CONSORT subitem 14a? *

Copy and paste relevant sections from the manuscript (include quotes in quotation marks "like this" to indicate direct quotes from your manuscript), or elaborate on this item by providing additional information not in the ms, or briefly explain why the item is not applicable/relevant for your study

Yes. "Assessments took place at baseline (T1), at post-treatment (seven weeks, T2), and at six months (T3; see Figure 1 for a detailed overview of assessments)."

14a-i) Indicate if critical "secular events" fell into the study period

Indicate if critical "secular events" fell into the study period, e.g., significant changes in Internet resources available or "changes in computer hardware or Internet delivery resources"

	1	2	3	4	5	
subitem not at all important	○	◉	○	○	○	essential

Auswahl löschen

Does your paper address subitem 14a-i?

Copy and paste relevant sections from the manuscript (include quotes in quotation marks "like this" to indicate direct quotes from your manuscript), or elaborate on this item by providing additional information not in the ms, or briefly explain why the item is not applicable/relevant for your study

No, not applicable since there were no secular events during the study period.

14b) Why the trial ended or was stopped (early)

Does your paper address CONSORT subitem 14b? *

Copy and paste relevant sections from the manuscript (include quotes in quotation marks "like this" to indicate direct quotes from your manuscript), or elaborate on this item by providing additional information not in the ms, or briefly explain why the item is not applicable/relevant for your study

No, not applicable since trial did not end early.

15) A table showing baseline demographic and clinical characteristics for each group

NPT: When applicable, a description of care providers (case volume, qualification, expertise, etc.) and centers (volume) in each group

Does your paper address CONSORT subitem 15? *

Copy and paste relevant sections from the manuscript (include quotes in quotation marks "like this" to indicate direct quotes from your manuscript), or elaborate on this item by providing additional information not in the ms, or briefly explain why the item is not applicable/relevant for your study

Yes. Ths is depicted in Table 1.

15-i) Report demographics associated with digital divide issues

In ehealth trials it is particularly important to report demographics associated with digital divide issues, such as age, education, gender, social-economic status, computer/Internet/ehealth literacy of the participants, if known.

	1	2	3	4	5	
subitem not at all important	○	○	○	◉	○	essential

Auswahl löschen

Does your paper address subitem 15-i? *

Copy and paste relevant sections from the manuscript (include quotes in quotation marks "like this" to indicate direct quotes from your manuscript), or elaborate on this item by providing additional information not in the ms, or briefly explain why the item is not applicable/relevant for your study

Yes. Demographics depicted in Table 1 include among others: Age, Gender, Marriage/ relationship status, Ethnicity Education, Working characteristics, Employment status, Working sector.

16) For each group, number of participants (denominator) included in each analysis and whether the analysis was by original assigned groups

16-i) Report multiple “denominators” and provide definitions

Report multiple “denominators” and provide definitions: Report N’s (and effect sizes) “across a range of study participation [and use] thresholds” [1], e.g., N exposed, N consented, N used more than x times, N used more than y weeks, N participants “used” the intervention/comparator at specific pre-defined time points of interest (in absolute and relative numbers per group). Always clearly define “use” of the intervention.

	1	2	3	4	5	
subitem not at all important	○	○	○	○	○	essential

Does your paper address subitem 16-i? *

Copy and paste relevant sections from the manuscript (include quotes in quotation marks "like this" to indicate direct quotes from your manuscript), or elaborate on this item by providing additional information not in the ms, or briefly explain why the item is not applicable/relevant for your study

Not applicable, since there were no multiple denominators.

16-ii) Primary analysis should be intent-to-treat

Primary analysis should be intent-to-treat, secondary analyses could include comparing only “users”, with the appropriate caveats that this is no longer a randomized sample (see 18-i).

	1	2	3	4	5	
subitem not at all important	○	○	○	◉	○	essential

Auswahl löschen

Does your paper address subitem 16-ii?

Copy and paste relevant sections from the manuscript (include quotes in quotation marks "like this" to indicate direct quotes from your manuscript), or elaborate on this item by providing additional information not in the ms, or briefly explain why the item is not applicable/relevant for your study

Yes.
"Therefore, following the intention-to-treat principle (ITT) missing data was handled with multiple imputations [66], using 100 estimations per missing value"

17a) For each primary and secondary outcome, results for each group, and the estimated effect size and its precision (such as 95% confidence interval)

Does your paper address CONSORT subitem 17a? *

Copy and paste relevant sections from the manuscript (include quotes in quotation marks "like this" to indicate direct quotes from your manuscript), or elaborate on this item by providing additional information not in the ms, or briefly explain why the item is not applicable/relevant for your study

Yes. Examplary for the primary outcome:
". The ANCOVA showed a significant group effect indicating that participants in the IG had, compared to the WLC, significantly lower scores on the PSS-10 at T2 (F1, 393=64.44, P<.001) and T3 (F1, 393=46.91, P<.001) with medium to large between-group-effect sizes at T2 (Cohen's d=0.71; 95%-CI: 0.51 to 0.91), and T3 (d=0.61; 95%-CI: 0.41 to 0.81)."

17a-i) Presentation of process outcomes such as metrics of use and intensity of use

In addition to primary/secondary (clinical) outcomes, the presentation of process outcomes such as metrics of use and intensity of use (dose, exposure) and their operational definitions is critical. This does not only refer to metrics of attrition (13-b) (often a binary variable), but also to more continuous exposure metrics such as "average session length". These must be accompanied by a technical description how a metric like a "session" is defined (e.g., timeout after idle time) [1] (report under item 6a).

	1	2	3	4	5	
subitem not at all important	○	○	○	●	○	essential

Auswahl löschen

Does your paper address subitem 17a-i?

Copy and paste relevant sections from the manuscript (include quotes in quotation marks "like this" to indicate direct quotes from your manuscript), or elaborate on this item by providing additional information not in the ms, or briefly explain why the item is not applicable/relevant for your study

Of the 198 participants in the IG, 188/198 (95.0%) finished session 1. Session 2 was completed by 171/198 (86.4%), session 3 by 154/198 (77.8%), session 4 by 135/198 (68.2%), session 5 by 121/198 (61.1%), session 6 by 108/198 (54.5%), session 7 by 94/198 (47.5%) and the booster session by 66/198 (33.3%) individuals.

17b) For binary outcomes, presentation of both absolute and relative effect sizes is recommended

Does your paper address CONSORT subitem 17b? *

Copy and paste relevant sections from the manuscript (include quotes in quotation marks "like this" to indicate direct quotes from your manuscript), or elaborate on this item by providing additional information not in the ms, or briefly explain why the item is not applicable/relevant for your study

Yes. Exemplary:
"Significantly less participants experienced a symptom deterioration in the IG (5.1%, 10/198) compared to WLC (9.6%;19/198), which reflects an absolute risk reduction of: 4.55% (95%-CI: -0.57%; 9.66%) and a relative risk reduction of 47.88% (95-CI: -10.31%; 74.89%)."

18) Results of any other analyses performed, including subgroup analyses and adjusted analyses, distinguishing pre-specified from exploratory

Does your paper address CONSORT subitem 18? *

Copy and paste relevant sections from the manuscript (include quotes in quotation marks "like this" to indicate direct quotes from your manuscript), or elaborate on this item by providing additional information not in the ms, or briefly explain why the item is not applicable/relevant for your study

Yes. Dedicated section about the moderator analyis.

Example: "Resilience moderated the intervention effect at T2 (P=.04) as well as at T3 (P=.01). The ROS at T2 and T3 ranged from 1 to 34.28, respectively 1 to 30.63 indicating that there was no significant between-group effect for participants with higher levels of resilience at baseline. In total 2.8% (11/396) of the sample had resilience scores > 34.28; 8.8% (35/396) had scores > 30.63."

18-i) Subgroup analysis of comparing only users

A subgroup analysis of comparing only users is not uncommon in ehealth trials, but if done, it must be stressed that this is a self-selected sample and no longer an unbiased sample from a randomized trial (see 16-iii).

	1	2	3	4	5	
subitem not at all important	○	◉	○	○	○	essential

Auswahl löschen

Does your paper address subitem 18-i?

Copy and paste relevant sections from the manuscript (include quotes in quotation marks "like this" to indicate direct quotes from your manuscript), or elaborate on this item by providing additional information not in the ms, or briefly explain why the item is not applicable/relevant for your study

Not applicable as it was not done.

19) All important harms or unintended effects in each group

(for specific guidance see CONSORT for harms)

Does your paper address CONSORT subitem 19? *

Copy and paste relevant sections from the manuscript (include quotes in quotation marks "like this" to indicate direct quotes from your manuscript), or elaborate on this item by providing additional information not in the ms, or briefly explain why the item is not applicable/relevant for your study

Yes. Symptom detoriation in both groups is described. Exemplary: "Significantly less participants experienced a symptom deterioration in the IG (5.1%, 10/198) compared to WLC (9.6%;19/198), which reflects an absolute risk reduction of: 4.55% (95%-CI: -0.57%; 9.66%) and a relative risk reduction of 47.88% (95-CI: -10.31%; 74.89%)."

19-i) Include privacy breaches, technical problems

Include privacy breaches, technical problems. This does not only include physical “harm” to participants, but also incidents such as perceived or real privacy breaches [1], technical problems, and other unexpected/unintended incidents. “Unintended effects” also includes unintended positive effects [2].

	1	2	3	4	5	
subitem not at all important	○	◉	○	○	○	essential

Auswahl löschen

Does your paper address subitem 19-i?

Copy and paste relevant sections from the manuscript (include quotes in quotation marks "like this" to indicate direct quotes from your manuscript), or elaborate on this item by providing additional information not in the ms, or briefly explain why the item is not applicable/relevant for your study

There were, luckily, no privacy breaches or technical problems.

19-ii) Include qualitative feedback from participants or observations from staff/researchers

Include qualitative feedback from participants or observations from staff/researchers, if available, on strengths and shortcomings of the application, especially if they point to unintended/unexpected effects or uses. This includes (if available) reasons for why people did or did not use the application as intended by the developers.

	1	2	3	4	5	
subitem not at all important	○	◉	○	○	○	essential

Auswahl löschen

Does your paper address subitem 19-ii?

Copy and paste relevant sections from the manuscript (include quotes in quotation marks "like this" to indicate direct quotes from your manuscript), or elaborate on this item by providing additional information not in the ms, or briefly explain why the item is not applicable/relevant for your study

Not direct feedback and observations obtained due to the design of the study (automated). Normal assesment of reasons in discussion, however.

DISCUSSION

22) Interpretation consistent with results, balancing benefits and harms, and considering other relevant evidence

NPT: In addition, take into account the choice of the comparator, lack of or partial blinding, and unequal expertise of care providers or centers in each group

22-i) Restate study questions and summarize the answers suggested by the data, starting with primary outcomes and process outcomes (use)

Restate study questions and summarize the answers suggested by the data, starting with primary outcomes and process outcomes (use).

	1	2	3	4	5	
subitem not at all important	○	○	○	○	○	essential

Does your paper address subitem 22-i? *

Copy and paste relevant sections from the manuscript (include quotes in quotation marks "like this" to indicate direct quotes from your manuscript), or elaborate on this item by providing additional information not in the ms, or briefly explain why the item is not applicable/relevant for your study

Yes.
"This study aimed to evaluate the effectiveness and moderators of treatment outcome of a self-guided iSMI as a universal prevention approach without an elaborative inclusion process based on baseline symptom severity. The results indicate that the training was highly effective in reducing stress levels in the short-term (d=0.71) and long-term (d=0.61) compared to a WLC. Significant moderate to large effects were found for several secondary mental health and work-related outcomes, including outcomes such as occupational self-efficacy for which no evidence of iSMIs was yet available. Moreover, this study is one of the first to investigate moderator effects in iSMIs in an adequately powered sample. The intervention was suitable for a wide range of participants. Only a few moderators of the intervention effect were identified indicating that employees with very high resilience, very low psychological strain, very high agreeableness, and very high self-regulation might not profit from the iSMI."

22-ii) Highlight unanswered new questions, suggest future research

Highlight unanswered new questions, suggest future research.

	1	2	3	4	5	
subitem not at all important	○	○	○	◉	○	essential

Auswahl löschen

Does your paper address subitem 22-ii?

Copy and paste relevant sections from the manuscript (include quotes in quotation marks "like this" to indicate direct quotes from your manuscript), or elaborate on this item by providing additional information not in the ms, or briefly explain why the item is not applicable/relevant for your study

Yes. Exemplary:
"Future studies should hence test whether the utilization of universal preventive approaches in employees can be further increased, for example by utilizing acceptance facilitating interventions [72–75] that are designed to reduce barriers of intervention utilization, such as low perceived risk."
""Future research should investigate if this was a spurious finding or could lead to a new understanding of the interaction between personality traits and iSMIs." and more.

20) Trial limitations, addressing sources of potential bias, imprecision, and, if relevant, multiplicity of analyses

20-i) Typical limitations in ehealth trials

Typical limitations in ehealth trials: Participants in ehealth trials are rarely blinded. Ehealth trials often look at a multiplicity of outcomes, increasing risk for a Type I error. Discuss biases due to non-use of the intervention/usability issues, biases through informed consent procedures, unexpected events.

	1	2	3	4	5	
subitem not at all important	○	○	○	●	○	essential

Auswahl löschen

Does your paper address subitem 20-i? *

Copy and paste relevant sections from the manuscript (include quotes in quotation marks "like this" to indicate direct quotes from your manuscript), or elaborate on this item by providing additional information not in the ms, or briefly explain why the item is not applicable/relevant for your study

Yes.
"This study also has some limitations to be considered. First, as usual with randomized-controlled trials, there was a screening process preceding trial participation, i.e., informed consent, which might have caused some applicants with lower motivation to drop out before study begin potentially resulting in a more homogenous sample. Moreover, the exclusion of suicidal individuals limits the generalizability to such samples. So, although this was a pragmatic trial, and eligibility criteria were kept to a minimum to mimic routine care, intervention effects might be overestimated compared to the intervention under routine conditions. Second, one needs to keep in mind that the evidence generated by this study is based on an RCT, which brings a rather high structuring of participants and a high research attention with it, which is usually not the case in routine occupational care. Since the securing of commitment represents an adherence-promoting element in self-help interventions, it can be assumed that the effect sizes for pure self-guided interventions found in RCTs are, significantly overestimated for what can be expected in occupational routine care [84] , when no additional measures to increase adherence are applied. Hence, a clear concept for ensuring adherence such as through minimal guidance from a professional seems favorable, especially when considering that guided Internet-based interventions have found to be superior over pure self-help interventions, both for stress-management [70] as well as other areas [85–87]. Preference should be in routine care, whenever possible, given to self-help approaches with at least some form of adherence-promoting guidance."

21) Generalisability (external validity, applicability) of the trial findings

NPT: External validity of the trial findings according to the intervention, comparators, patients, and care providers or centers involved in the trial

21-i) Generalizability to other populations

Generalizability to other populations: In particular, discuss generalizability to a general Internet population, outside of a RCT setting, and general patient population, including applicability of the study results for other organizations

	1	2	3	4	5	
subitem not at all important	○	○	○	◉	○	essential

Auswahl löschen

Does your paper address subitem 21-i?

Copy and paste relevant sections from the manuscript (include quotes in quotation marks "like this" to indicate direct quotes from your manuscript), or elaborate on this item by providing additional information not in the ms, or briefly explain why the item is not applicable/relevant for your study

Yes. Due to the research question, this is a main focus of the discussion and is also critically reflected within limitations.

21-ii) Discuss if there were elements in the RCT that would be different in a routine application setting

Discuss if there were elements in the RCT that would be different in a routine application setting (e.g., prompts/reminders, more human involvement, training sessions or other co-interventions) and what impact the omission of these elements could have on use, adoption, or outcomes if the intervention is applied outside of a RCT setting.

	1	2	3	4	5	
subitem not at all important	○	○	○	◉	○	essential

Auswahl löschen

Does your paper address subitem 21-ii?

Copy and paste relevant sections from the manuscript (include quotes in quotation marks "like this" to indicate direct quotes from your manuscript), or elaborate on this item by providing additional information not in the ms, or briefly explain why the item is not applicable/relevant for your study

Yes, see answer before. Due to the research question, this is a main focus of the discussion and is also critically reflected within limitations.

OTHER INFORMATION

23) Registration number and name of trial registry

Does your paper address CONSORT subitem 23? *

Copy and paste relevant sections from the manuscript (include quotes in quotation marks "like this" to indicate direct quotes from your manuscript), or elaborate on this item by providing additional information not in the ms, or briefly explain why the item is not applicable/relevant for your study

Yes. "German Register of Clinical Studies (DRKS)"

24) Where the full trial protocol can be accessed, if available

Does your paper address CONSORT subitem 24? *

Cite a Multimedia Appendix, other reference, or copy and paste relevant sections from the manuscript (include quotes in quotation marks "like this" to indicate direct quotes from your manuscript), or elaborate on this item by providing additional information not in the ms, or briefly explain why the item is not applicable/relevant for your study

There was no trial protocol specifically for this trial but for the "sister trial" which focussed on stressed employees. This is cited in the manuscript "(Heber et al., 2013)" - Efficacy and cost-effectiveness of a web-based and mobile stressmanagement
intervention for employees: design of a randomized controlled trial

25) Sources of funding and other support (such as supply of drugs), role of funders

Does your paper address CONSORT subitem 25? *

Copy and paste relevant sections from the manuscript (include quotes in quotation marks "like this" to indicate direct quotes from your manuscript), or elaborate on this item by providing additional information not in the ms, or briefly explain why the item is not applicable/relevant for your study

Yes.
"We would like to acknowledge the European Union (EFRE) for funding this project within the Lueneburg Innovation Incubator, TM 1.1 (project number: CCI 2007DE161PR001)."

X27) Conflicts of Interest (not a CONSORT item)

X27-i) State the relation of the study team towards the system being evaluated

In addition to the usual declaration of interests (financial or otherwise), also state the relation of the study team towards the system being evaluated, i.e., state if the authors/evaluators are distinct from or identical with the developers/sponsors of the intervention.

	1	2	3	4	5	
subitem not at all important	○	◉	○	○	○	essential

Auswahl löschen

Does your paper address subitem X27-i?

Copy and paste relevant sections from the manuscript (include quotes in quotation marks "like this" to indicate direct quotes from your manuscript), or elaborate on this item by providing additional information not in the ms, or briefly explain why the item is not applicable/relevant for your study

Meine Antwort

About the CONSORT EHEALTH checklist

As a result of using this checklist, did you make changes in your manuscript? *

- ○ yes, major changes
- ○ yes, minor changes
- ◉ no

What were the most important changes you made as a result of using this checklist?

Double-checked that everything is included.

How much time did you spend on going through the checklist INCLUDING making changes in your manuscript *

45min-1h (re-used checlist form initial submission as guidance)

As a result of using this checklist, do you think your manuscript has improved? *

- ○ yes
- ○ no
- ◉ Sonstiges: slightly, since it was already written with CONSORT in mind.

Would you like to become involved in the CONSORT EHEALTH group?

This would involve for example becoming involved in participating in a workshop and writing an "Explanation and Elaboration" document

- ○ yes
- ◉ no
- ○ Sonstiges:

Auswahl löschen

Any other comments or questions on CONSORT EHEALTH

CONSORT is great for authors and readers.

STOP - Save this form as PDF before you click submit

To generate a record that you filled in this form, we recommend to generate a PDF of this page (on a Mac, simply select "print" and then select "print as PDF") before you submit it.

When you submit your (revised) paper to JMIR, please upload the PDF as supplementary file.

Don't worry if some text in the textboxes is cut off, as we still have the complete information in our database. Thank you!

Final step: Click submit !

Click submit so we have your answers in our database!

Senden

Geben Sie niemals Passwörter über Google Formulare weiter.

Dieser Inhalt wurde nicht von Google erstellt und wird von Google auch nicht unterstützt. Missbrauch melden - Nutzungsbedingungen - Datenschutzerklärung

Google Formulare
